# Habitat characteristics that favour the presence of *Aedes aegypti* (Diptera: Culicidae) in households in the city of Córdoba, a temperate area of Argentina

**DOI:** 10.1186/s13071-025-07114-1

**Published:** 2025-11-25

**Authors:** Carola Soria, Liliana Beatriz Crocco, Marta Gladys Grech, Anna Stewart-Ibarra, Walter Ricardo Almirón

**Affiliations:** 1 Cátedra de Introducción a la Biología, Facultad de Ciencias Exactas, Físicas y Naturales, Universidad Nacional de Córdoba, Córdoba, Argentina; 2 Instituto de Investigaciones Biológicas y Tecnológicas- Consejo Nacional de Investigaciones Científicas y Técnicas (IIBYT-CONICET), Córdoba, Argentina; 3 Centro de Investigación Esquel de Montaña y Estepa Patagónica–Consejo Nacional de Investigaciones Científicas y Técnicas (UNPSJB-CONICET), Esquel, Chubut Argentina; 4 Universidad Nacional de La Patagonia San Juan Bosco, Esquel, Chubut Argentina; 5 Inter-American Institute for Global Change Research (IAI), Panama City, Panama

**Keywords:** Artificial breeding sites, Microenvironmental variables, Environmental variables, Peridomiciles

## Abstract

**Background:**

*Aedes aegypti* is the vector of dengue fever and chikungunya in the city of Córdoba, Argentina, a city situated at the southern limit of disease transmission by the vector in a temperate area. This study aims to characterise the habitat of juvenile *Ae. aegypti* in households and assess the influence of microenvironmental and environmental variables on its occurrence.

**Methods:**

Monthly surveys were conducted from 2019 to 2020 and in 2021 in peridomiciles. Water containers where *Ae. aegypti* were found were classified according to their material, class and capacity. Using generalised linear mixed models, we evaluated the influence of surrounding vegetation, water container availability and weather conditions (air temperature and precipitation during autumn, summer and spring) on the presence and abundance of juvenile *Ae. aegypti.*

**Results:**

Of the 689 containers surveyed, 109—found in 36.7% (77/210) of the households—contained juvenile *Ae. aegypti*. Small (1–8 l) and very small (< 0.5 l) containers accounted for 80.4% of the containers positive for juvenile *Ae. aegypti*, with plastic jars being the most common. Vases and tarpaulins/plastic covers were the most productive containers. Containers shaded by vegetation were 2.5-fold more likely to harbour juveniles than those exposed to sunlight or artificial shade. The strong interaction between tree cover and shade provided by vegetation suggested that vegetation cover enhances mosquito abundance. Juvenile abundance increased with container capacity, while higher precipitation and minimum temperature in the previous weeks also favoured the presence of juveniles.

**Conclusions:**

Household containers, which are widely available and diverse in size and material in Córdoba City, provide favourable conditions for the persistence of *Ae. aegypti*. Abandoned nonfunctional and cryptic sites, such as tarpaulins and plastic covers, can provide breeding sites that enable *Ae. aegypti* mosquitoes endure the cold and dry seasons. During the warm and wet seasons, the presence and abundance of *Ae. aegypti* are influenced by microenvironmental conditions, such as shade provided by vegetation, high minimum temperatures and precipitation. The variety and number of available containers, together with shelter and feeding conditions, suggest that female mosquitoes can still find breeding sites even after selective household control measures have been implemented. These results, supported by generalised linear mixed models, which consider the lack of independence between containers within the same household or block, highlight the importance of incorporating fine-scale environmental variables into vector control planning.

**Graphical Abstract:**

**Figure:**
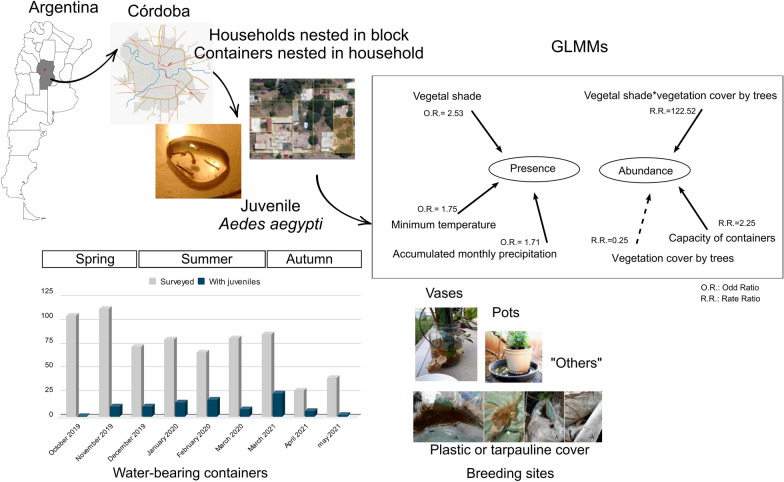

**Supplementary Information:**

The online version contains supplementary material available at 10.1186/s13071-025-07114-1.

## Background

*Aedes aegypti* (Diptera: Culicidae) is a synanthropic mosquito with the ability to transmit emerging and endemic arboviruses such as dengue, Zika and chikungunya [1]. The global distribution of *Ae. aegypti* is influenced mainly by climatic conditions. Consequently, climate change can alter the geographic distribution of the vector, the risk of disease outbreaks in new regions and the magnitude of outbreaks in endemic regions [2, 3]. Currently, the distribution pattern is concentrated in global tropical and subtropical regions and extends into temperate regions [4].

 In 2024, 12,673,750 cases of dengue were reported in Latin America. Specifically, in the temperate Southern Cone, a geographical subregion of South America and a zone of disease emergence, there was an increase of 262% compared with that in 2023 and an increase of 434% compared with the average of the preceding 5 years [5]. Dengue fever emerged in the city of Cordoba, Argentina, which has a temperate climate, for the first time in 2009, and since then, new outbreaks have been recorded successively [6]. Chikungunya fever cases have also been reported [7]. Preventing the emergence of arboviruses critically depends on understanding vector ecology and designing targeted public health interventions to reduce vector populations and interrupt virus transmission [8, 9]. This strategy will likely remain a priority even when a dengue vaccine becomes available [9].

Immature *Ae. aegypti* develop in natural habitats such as tree holes and leaf axils, as well as in diverse artificial breeding sites [10], such as tyres, bottles, buckets and any other water-holding container commonly found in household peridomiciles [11]. *Aedes aegypti* is highly anthropophilic; adults usually fly between 10 and 30 m from breeding sites, but pupae do not cluster beyond the household [12, 13], and females spend their lives in or near the household where they emerge [12, 14]. A recent global review indicated that the average maximum travel distance for an adult *Ae. aegypti* is approximately 100 m [15]. Accordingly, households are the entomological unit and the epidemiological surveillance unit for arbovirus transmission, and the block corresponds to the likely maximum travel distance of *Ae. aegypti*. Additionally, for the city of Córdoba, spatiotemporal cluster analysis of the 2009 dengue outbreak revealed that human mobility mediates the spread of the virus [16].

*Aedes aegypti* adult females often oviposit in several water-holding containers in a single household [17], and dispersal distances of females decrease as the availability of suitable oviposition sites increases [18]. Thus, social factors that result in high numbers of suitable oviposition sites may result in clusters of *Ae. aegypti* in the same household [14], favouring the persistence of this vector in the peridomicile.

In anthropised environments, the distribution and spatial orientation of food sources, physical refuges and the edge of vegetation determine the heterogeneous distribution of vectors over distances of just a few metres [19]. Therefore, information on the characteristics surrounding breeding habitats is crucial for designing specific vector control approaches for disease prevention. [20]. The different types, materials and capacities of artificial containers represent differential success as breeding environments for *Ae. aegypti* [21, 22]. In addition, microhabitat characteristics influence the suitability of containers as breeding sites for *Ae. aegypti* [23]. For example, containers located near vegetation may represent a more favourable microhabitat for *Ae. aegypti* [24, 25] because of the mosquito’s preference to rest in the shade before oviposition, the sugar feeding behaviour of adults, the increased nutrient supply for juvenile mosquitoes related to vegetation falling into the container and the thermal regulation of water in the container [26].

Although *Ae. aegypti* has been studied extensively around the world, the specific characteristics that favour its presence vary over time and between localities [27]. In the city of Córdoba (Argentina), the location of this study, *Ae. aegypti* population dynamics are strongly bounded by low temperatures, with no oviposition or embryonic development during the cold winter months [28, 29]. Additionally, the developmental time of immature stages is inversely influenced by temperature and precipitation [30]; specifically, the preadult developmental time of *Ae. aegypti* varies depending on the season [28]. In summer, when the average temperature in Córdoba is 22 °C, total preadult developmental time has been reported to be 16.12 days; in autumn, with an average temperature of 21 °C, the preadult developmental time increased to 18.10 days; during spring, with an average temperature of 19 °C, the total preadult developmental time reached 25.88 days. Also, precipitation affects the *Ae. aegypti* juvenile population with a 4-week lag [28].

In the city of Córdoba, more urbanised areas with higher population density and proximity to vegetated zones have been associated with increased mosquito infestation levels and oviposition activity [31–33]. However, these studies describe within-city patterns and are performed at aggregated spatial scales (neighbourhoods or a network of traps spaced several hundred metres apart). There is a knowledge gap regarding the ecology of *Ae. aegypti* at a fine spatial scale, specifically within individual households, which are considered to be the entomological and epidemiological units of analysis. Moreover, in households, social and ecological variables interact and influence the breeding potential of mosquitoes. Therefore, the aim of this work was to characterise *Ae. aegypti* artificial breeding sites and assess the impacts of surrounding vegetation, water-holding container availability and weather conditions (air temperature and precipitation during the autumn, summer and spring) on the presence and abundance of juvenile *Ae. aegypti* at the household scale.

## Methods

### Study area

**Fig. 1:**
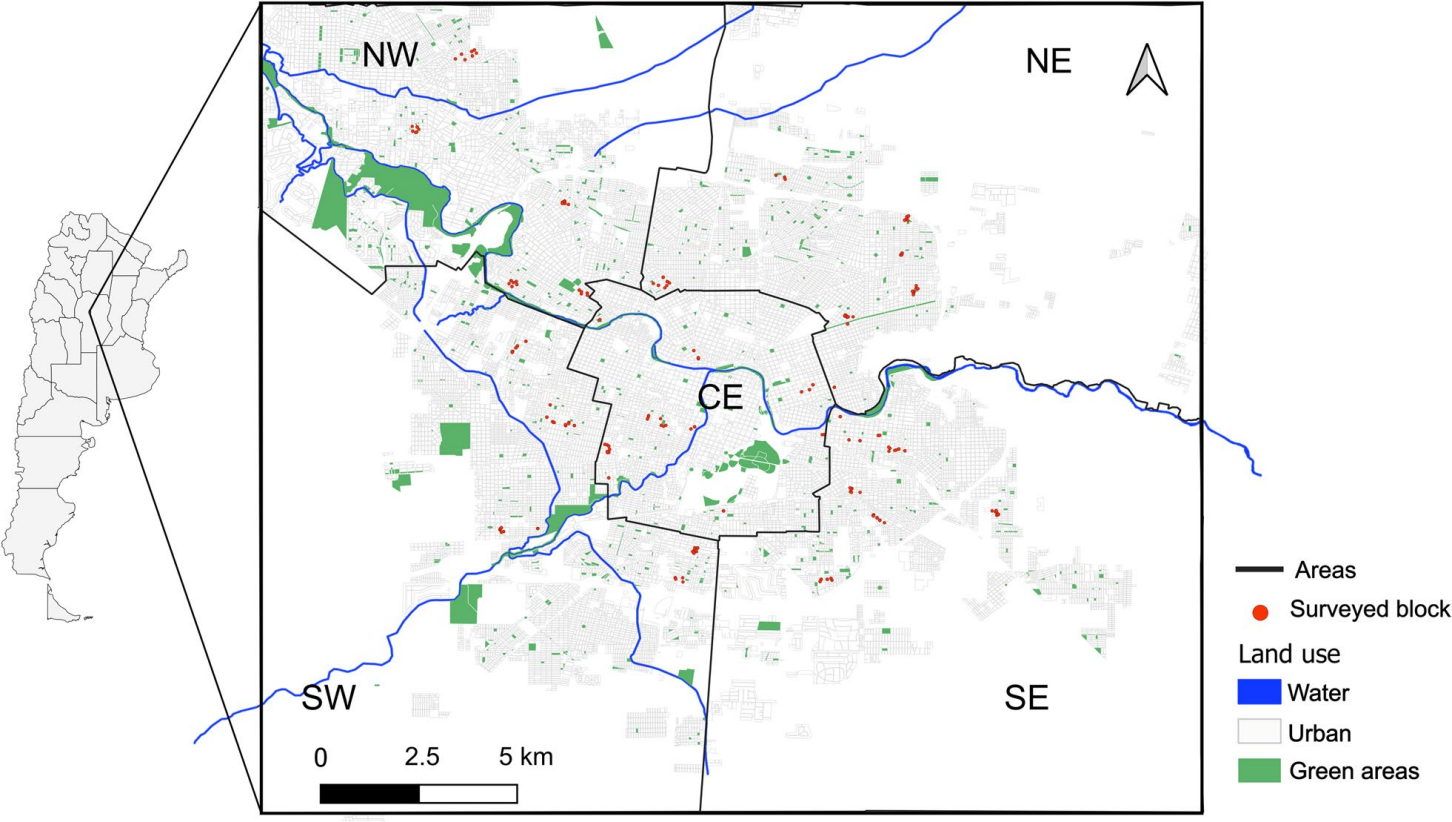
Map of Córdoba city (Córdoba Province, Argentina), divided into five areas according to the *Aedes aegypti* surveillance programme of the Epidemiology Department (Ministry of Health of Cordoba). CE, Centre; NE, northeast; NW, northwest; SE, southeast; SW,  southwest

The work was conducted in the city of Córdoba (31°24′S, 64°11′W), Argentina (Fig. 1). The city is located in a temperate zone, with a rainy and warm season from October to May (average daily minimum and maximum temperatures of 16.2 °C and 28.4 °C, respectively, and average monthly precipitation of 105.5 mm), and a dry and cold season from June to September (average daily minimum and maximum temperatures of 7.8 °C and 20.8 °C, respectively, and average monthly precipitation of 15.6 mm) [34].

The city of Córdoba is the capital of Córdoba Province and is the second largest city in Argentina. It is a major urban centre with a total area of 573.2 km^2^ and a population of 1,498,060 inhabitants [35]. The urban structure has a highly developed central core where government, commercial and residential activities are concentrated. Surrounding this core, in a radiocentric pattern, is the peripheral area, with residential neighbourhoods that co-exist with areas of other land uses. A beltway encircles these areas, delimiting an outward urban expansion zone, which also includes production and industrial areas and the San Martín Urban Nature Reserve in the north-western area (Fig. 1; Additional file 1: Figure S1). The Suquía River, its tributary La Cañada and additional drainage channels flow through the city (Fig. 1). The description of land use categories within the study area was based on cartographic data provided by the Spatial Data Infrastructure of the Province of Córdoba (IDECOR) (https://mapascordoba.gob.ar/). The coverage of essential services follows a gradient, with maximum levels in the centre and decreasing towards the periphery. Over 90% of households are connected to the public sewage network and have piped drinking water supplies [35]. The term peridomicile is applied to an outdoor space surrounding the indoor area, bounded on all sides and open to the sky.

### Sampling design

Field studies were conducted within the framework of the *Ae. aegypti* surveillance programme of the Department of Epidemiology of the Cordoba Province Ministry of Health in cooperation with the Cordoba National University (UNC) and the Biological and Technological Research Institute (IIByT) of National Research Council (CONICET). This design was implemented in Córdoba through the surveillance programme in 2009 and continues up to the present [29]. Technicians from the Ministry of Health were trained to follow standard protocols for vector surveillance. Data collection was conducted with the verbal consent of household residents, in accordance with public health guidelines. The Ministry of Health informed the population that weekly monitoring would be carried out in selected areas of the city. These announcements were intended to ensure that residents were aware of the scheduled visits by surveillance technicians.

The surveillance programme involves the monthly sampling of larvae and pupae, conducted between October and May because *Ae. aegypti* does not oviposit during the cool temperatures of the winter and the vectors persist through the cold season as eggs in Córdoba [29, 30]. Monitoring between October and May is aligned with the rainy and warm season in Córdoba, when conditions favour egg hatching, juvenile and adult development and an increase in *Ae. aegypti* populations [29]. For operational purposes, the surveillance programme subdivides Córdoba into a grid of 215 squares (1.2 km per side), grouped into five areas: the Central, Northeast, Southeast, Northwest and Southwest areas (Fig. 1). The Central area (CE) is the smallest unit, forming the urban core with high population and household density. The Northeast (NE) and Southeast (SE) are both large urban areas, with moderate population density, minimal green space and the largest share of industrial, commercial and other non-residential land. The Northwest (NW) is an area with a relatively lower population density, reflecting suburban expansion, and includes the San Martín Urban Nature Reserve, a 1.4-km^2^ green area. The Southwest (SW) contains mostly vacant land or land undergoing urbanisation, with a population density higher than that of other peripheral zones (Additional file 1: Figure S1; Additional file 2: Table S1).

Each month, between October 2019 and March 2020 (season 1), one grid was randomly selected from each of the different areas (CE, NE, NW, SE and SW) by the research team. Sampling occurred over 5 consecutive days, with one grid from a different area visited each day. This approach provided monthly coverage of all areas, maintaining spatial representation. Technicians from the Ministry of Health were provided with maps of all households within each grid and followed a predefined route. Twenty households per grid were selected, starting at a designated household and continuing to successive households; if there was no response, the next household was approached, and the procedure was repeated until access had been obtained to 20 households. In each of the selected household, residents were invited to respond to an interview on their knowledge and practices related to *Ae. aegypti* prevention. Afterwards, educational leaflets on dengue and the vector were provided, and residents were asked for permission to inspect water containers and record the characteristics of peridomiciles in their backyards. As most houses in the city are closed at the front, the presence of containers could not be observed from the street; consequently, technicians had no prior knowledge of whether or not containers were present. A second sampling season independent of the surveillance programme, which was interrupted due to the COVID-19 pandemic, was conducted between March and May 2021. In the second season, at least two households were sampled along a 1.2-km-diameter grid to simulate the sampling of the previous season. The research team personally contacted the residents beforehand to arrange a visit, and residents were informed about the purpose of the survey; prior to entry by the research team, residents provided formal consent. All of the households visited in both seasons were located in areas of in urban land use (Fig. 1) and were georeferenced using a Global Positioning System (GPS).

### Response and explanatory variables

Containers with water found in the peridomiciles were visually inspected for juvenile *Ae. aegypti*. Containers positive for the presence of juveniles were considered to be breeding sites. Subsequently, a live sample of juveniles from each container was transported to the laboratory for rearing purposes and to identify the mosquito species. In the second sampling season, all water in a container that tested positive for juvenile *Ae. aegypti* was transferred to the laboratory to identify and count larvae and/or pupae. Water in juvenile *Ae. aegypti*-positive containers with a volume > 2 l was filtered through a plastic mesh, and the live larvae and pupae were manually extracted and transported to the laboratory. The second- and third-instar larvae were fed bovine (*Bos taurus*) liver powder until they reached the fourth instar for species identification [36]. Pupae were reared until adult emergence. All individual mosquitoes were morphologically identified following the key by Darsie [37]; however, larvae identified as *Culex quinquefasciatus* are referred to here as the *Culex pipiens* complex since recent studies have indicated that *Cx. pipiens* and hybrids can also be found in Córdoba [38].

We recorded the number of water-holding containers per peridomicile, classifying the containers by material, class and capacity (volume in litres of water it may hold). We recorded whether containers were exposed to the sun, shaded by vegetation (under vegetation canopy) or shaded by a constructed roof (under a man-made artificial structure) at the time of the survey (between 9.00 a.m. and 1.00 p.m.).

The capacity of the containers was assessed using complementary methods. For containers with a labelled or standardised size (e.g. buckets and tanks), the nominal value indicated by the manufacturer was recorded. For unlabelled containers, the capacity was determined in situ by filling the container with water, using a graduated container as a reference, immediately after sampling larvae and pupae. In the case of fixed structures, such as drains, the capacity was estimated by measuring their maximum internal dimensions (length, width and total depth) and applying the volumetric formula of the closest geometric solid. For water found in tarpaulins or plastic bags, where water accumulated in folds or shallow depressions, the capacity was determined by extracting the volume of water with a graduated Pasteur pipette and recording the total amount of liquid recovered.

Using the georeferenced data recorded for each household, a reference map (1:700) was generated in the QGIS Geographic Information System (http://www.qgis.org), incorporating the cadastral parcel layer provided by the Córdoba City cadastre (https://mapascordoba.gob.ar/) along with satellite images from Google Earth. The households visited had an average (± standard deviation (SD) plot size of 0.00025 ± 0.000114 km^2^ (250 ± 114 m^2^). For each plot, the backyard area was estimated by subtracting the built-up area from the total parcel area, based on quantitative cadastral information. Backyard sizes ranged from 32 to 212 m^2^, with a mean and median of 136 and 154 m^2^, respectively; 75% of the backyards were smaller than 180 m^2^. Vegetation cover within each backyard was then quantified using the QGIS area calculation tool, based on high-resolution imagery from Google Earth. The proportion of vegetation cover was calculated as the area of vegetation cover relative to the total backyard area. During household visits, vegetation was classified as herbaceous/shrub cover (< 1.5 m in height) or tree cover (> 1.5 m in height).

The average daily temperature (°C) and precipitation (mm) data were provided by Córdoba Observatorio meteorological stations operated by the National Meteorological Service (SMN) [34]. Daily temperatures (minimum, mean and maximum) were obtained for the 20 days prior to the date of collection in the spring season, for the 15 days prior to the date of collection in the summer season and for the 12 days prior to the date of collection in the autumn season; these time periods are the time required to detect larvae and/or pupae of *Ae. aegypti* in the containers after embryonic development in the city of Córdoba according to Domínguez et al. [28] and Grech et al. [30]. Additionally, accumulated precipitation during the 30 days prior to the collection day of larvae and/or pupae was recorded. All of the variables considered for this study are summarised in Table 1.

**Table 1:** Variables used to characterise habitats that favour the presence and abundance of juvenile *Aedes aegypti* around households in the city of Córdoba, Argentina

Variables	Variable specifics	Definition
Container	Class (qualitative)	Animal water dishes, bottles, buckets, canvas pools, drains, dishes, flower pots, jars, plastic shopping bags, plastic tarp with collected rainwater, swimming pools, tyres, water tanks and others
	Material (qualitative)	Cement, ceramic, glass, metal, plastic and rubber
	Capacity (quantitative)	Maximum potential container capacity in litres
	Capacity (qualitative)	Very small (< 0.5 l), small (1—8 l), medium (10—20 l), large (50—200 l), very large (1000–5,000 l), extremely large (> 25,000 l)
	Shade (qualitative)	Artificial shade projected by a built roof (shaded by artificial structures), vegetal shade (shaded by vegetation); none (fully exposed to sunlight)
Peridomicile	Containers with water	Number of containers with water
	Vegetation cover by herbs and shrubs	Proportion of surface cover by herbs and shrubs (> 0.5 m and < 1.5 m)
	Vegetation cover by trees	Proportion of surface cover by trees (> 1.5 m)
Weather conditions	Minimum, maximum and mean temperature	Minimum, maximum and mean daily temperatures recorded for 20 days prior to the collection of larvae and/or pupae during spring season, 15 days prior during the summer season and 12 days prior during the fall season
	Monthly accumulated precipitation	Accumulated precipitation recorded during the 30 days prior to the collection day of juveniles
Response	Presence of juvenile *Aedes aegypti* (qualitative)	
	Abundance of juvenile *Aedes aegypti* (quantitative)	

### Data analysis

The Container Index (CI) was calculated as the percentage of inspected containers with *Ae. aegypti* [(number of containers with *Ae. aegypti*/number of containers inspected) × 100]. The Household Index (HI) was calculated as the percentage of inspected houses with at least one container with *Ae. aegypti* [(number of houses with at least one container with *Ae. aegypti*/number of houses inspected) × 100].

A proportion test was applied to evaluate the relationship between the number of breeding sites and the total number of water-holding containers found, each of which was classified by material and class variables [39]. The remaining quantitative explanatory variables were standardised prior to the analysis, with a mean of zero and a standard deviation (SD)equal to 1. Analyses were conducted using R [40] in the RStudio environment [41]. The correlations among explanatory quantitative variables were evaluated in the corrplot package [42], and a threshold of ≥ 0.70 was set for the correlation coefficient. To check for spatial autocorrelation among containers, Moran’s index was estimated via a Euclidean distance matrix with the APE package [43]. Because a slight spatial structure was evident (Moran’s index = 0.158,* p* =  < 0.001), all areas of the city were considered to be equally represented. The relationships between response variables and explanatory variables were analysed with generalised linear mixed models (GLMMs) [44] via the glmmTMB function of the glmmTMB package [45]. To account for the hierarchical sampling structure, as proposed by Flaibani et al. [46], the presence models included random effects for containers nested within households and households nested within blocks. The abundance models included random effects for containers nested within households because only one household per block was surveyed.

To assess the presence of juvenile *Ae. aegypti* in containers (1 = breeding sites, 0 = containers without juveniles), GLMMs with a binomial distribution and logit link function were used [44]. Full models including all explanatory variables, with or without interactions between the “shade” and “vegetation cover” variables, were compared to a null model that included only the random factors [47]. This comparison was performed using the likelihood ratio test [48], implemented through the analysis of variance (ANOVA) function of the CAR package [49]. The model without interaction was different from the null model (Additional file 3: Table S2; Additional file 4: Table S3). To avoid redundancy, the minimum, maximum and mean daily temperatures were analysed separately in different models, including all the remaining independent variables. The minimum temperature model showed the lowest Akaike information criterion (AIC) [50], which was at least 2 units of delta AIC different from the other models, according to comparisons calculated with the ‘model.sel’ function of the ‘MuMIn’ package [51] (Additional file 4: Table S3). The residuals were evaluated graphically with the broom [52] and DHARMa [53] packages, including normality, residual patterns against fitted values and explanatory variables and influential data points.

A Poisson GLMM was used to identify the explanatory variables associated with the abundance of juveniles of *Ae. aegypti,* [44]. No zero inflation was detected, and the model showed a good fit, with an adequate distribution of residuals, no evidence of overdispersion and no significant patterns in the residuals versus the predicted values (Additional file 5: Figure S2, Additional file 6: Figure S3). These tests were performed with the DHARMa package [53]. Full models including all explanatory variables, with or without interactions between the “shade” and “vegetation cover” variables, were compared to a null model including only the random factors [47]. A likelihood ratio test was used [48], implemented through the ANOVA function from the CAR package [49]. The model with interaction was significantly different from the null model (Additional file 7: Table S).

Finally, models of juvenile presence and abundance were selected via the "drop1" function [54]. The terms were removed one by one until a final model was reached, in which all the significant terms were obtained. The levels of qualitative variables were compared post hoc via the Tukey test with a significance level of 0.05. The significance of the coefficient of regression was tested using the Wald test. Model accuracy for the final model of the presence of *Ae. aegypti* was measured with the area under the curve (AUC) function via the ROCR package [55].

## Results

Across the two sampling seasons, a total of 689 water-holding containers were found in the 210 peridomiciles inspected. A total of 109 containers (15.82% of all containers) were positive for juvenile *Ae. aegypti* (72 containers in season 1, 37 in season 2)*.* These containers were located in 36.7% (77/210) of the peridomiciles. The distribution of containers and households surveyed across areas and the Container and Household indices are summarised in Table 2 and Additional file 8: Table S5.

**Table 2:** *Aedes aegypti* Container Index and Household Index across five areas in the city of Córdoba, Argentina, between October 2019 and March 2020 and between March and May 2021

Area of Córdoba City	Number of containers surveyed	Container Index^a^	Number of households surveyed	Household Index^b^
Central	103	19.42	40	32.5
Northeast	145	9.66	46	23.9
Northwest	153	17.65	49	36.7
Southeast	197	14.72	47	46.8
Southwest	91	20.88	28	46.4

^a^Container Index was calculated as the percentage of inspected containers with *Ae. aegypti*: [(number of containers with *Ae. aegypti*/number of containers inspected) × 100]

^b^Household Index was calculated as the percentage of inspected houses with at least one container with *Ae. aegypti*: [(number of houses with at least one container with *Ae. aegypti*/number of houses inspected) × 100]

During the second sampling season, when all the juveniles were collected and counted, a total of 713 larvae and 48 pupae were collected from the 37 positive containers (Table 3).

**Table 3:** Percentages of juvenile *Aedes aegypti* according to larval and pupal stage collected in the city of Córdoba between March and May 2021

	Larval instar	Percentage of total	Percentage of total larvae	Number of individuals
Larvae	–	93.7	–	713
	Larvae II	8.3	8.84	63
	Larvae III	61.6	65.8	469
	Larvae IV	23.8	25.4	181
Pupae	–	6.3	–	48

**Fig. 2:**
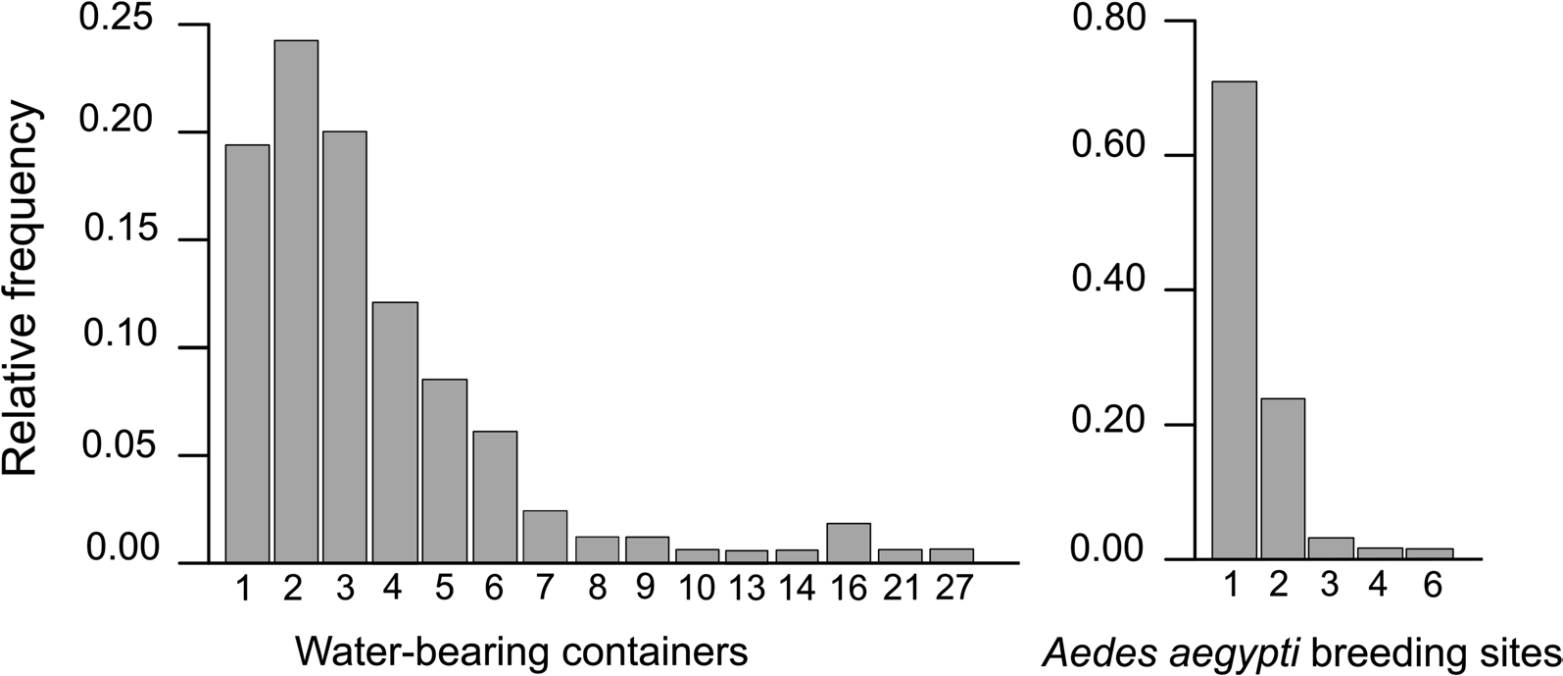
Relative frequency of water-holding containers (*n* = 684) and breeding sites of *Aedes aegypti* (*n* = 109) in peridomiciles of the city of Córdoba, Argentina, between October 2019 and March 2020 and between March and May 2021

Each peridomicile had between one and 27 water-holding containers, whereas the average number of positive containers was 3.56 (range 1–6) (Fig. 2). During the survey in season 2, the average number of juvenile *Ae. aegypti* counted per container was 5.9 (median 0, SD 20.6, range 0–148), and the average number per peridomicile was 35 (median 14, SD 42.5, range 0–158).

Juvenile *Culex* spp. co-occurred with *Ae. aegypti* in 4.6% (5/109) of the containers positive for *Ae. aegypti*. In two containers, *Culex* spp. were identified as members of the *Cx. pipiens* complex. We found that 3.6% (25/689) of all the water-holding containers contained only juvenile *Culex* spp.; of these 25 containers, 32% (*n* = 8) contained specimens from the *Cx. pipiens* complex and 8% (2/25) contained *Culex apicinus;* the rest were identified only to the genus level.

Plastic containers were the most common habitat for juvenile *Ae. aegypti* (53.57%; 58/109), followed by glass (14.29%; 16/109), metal (11.61%; 13/109), ceramic (8.93%; 10/109), cement (5.36%; 6/109), and rubber (3.57%; 4/109) containers. Plastic and glass containers accounted for 79% of the juveniles identified. In addition, jars represented the highest overall percentage of juvenile *Ae. aegypti*-positive habitats when these habitats were classified according to container class (23.21%; 25/109). However, vases, tarpaulins/plastic covers, pots and “others” were more likely to be positive for juvenile *Ae. aegypti* when they were present (e.g. number of positive containers per number of available containers of a given class) (Table 4). In addition, during the second sampling season, the highest mean (± SD) abundance of juveniles per container was found in inspected vases and tarpaulins/plastic covers (41.86 ± 57.14 and 22.33 ± 49.06, respectively) (Table 5). The breeding sites classified as “others” were specific uncommon containers, such as: ( i) an upside-down plastic garden chair that collected water from the armrests; (ii) a ceramic toilet fixture; (iii) watering cans; (iv) the trailer of a plastic toy truck; (v) an ornamental water fountain; and (vi) a plastic corner of a temporary plastic pool. In one peridomicile, no artificial breeding sites were detected, but the presence of *Ae. aegypti* adults led researchers to inspect natural containers, resulting in the collection of larvae and one pupa from the apical leaf axil of *Callisia fragrans* (Commelinaceae). The frequency distribution of surveyed water containers and *Ae. aegypti* breeding sites, stratified by container class and city area is shown in Additional file 9: Figure S4 .

**Table 4:** Containers with juvenile *Aedes aegypti* classified according to material, class, capacity, month of survey and type of shade recorded between October 2019 and March 2020 and between March and May 2021 in the city of Córdoba, Argentina

Containers—descriptive variables	Number of containers surveyed	Container Index^a^
*Material*		
Plastic	387	15.50
Glass	145	11.03
Metal	62	20.97
Cement	56	10.71
Ceramic	18	55.56
Rubber	16	25.00
Vegetal	5	60.00
*Class*		
Apical leaf axil	5	60.00
Bottles	134	5.22
Jars	123	21.14
Buckets	108	12.96
Animal water containers	72	4.17
Dishes	53	9.43
Vases^b^	44	31.82
Floor drains	43	16.28
Pots^b^	23	39.13
Plastic or tarpaulin covers^b^	17	47.06
Others^b^	17	47.06
Temporal plastic pools	17	11.76
Tyres	15	26.67
Pools	13	7.69
Tank	5	20.00
*Capacity (litres)*		
Very small (< 0.5)	167	18.56
Small (1—8)	399	12.03
Medium (10—20)	91	15.38
Large (50—200)	4	25.00
Very large (1000 a 5000)	13	15.38
Extremely large (> 25,000)	10	10.00
*Month and year of survey*		
October 2019	110	0.93
November 2019	134	9.65
December 2019	84	14.86
January 2020	82	18.29
February 2020	68	26.47
March 2020	83	9.64
March 2021	64	28.74
April 2021	33	21.43
May 2021	31	4.88
*Shade*		
Vegetation	83	32.53
Artificial	182	17.58
None (sun)	255	14.9

^a^Container Index was calculated as the percentage of inspected containers with *Ae. aegypti*: [(number of containers with *Ae. aegypti*/number of containers inspected) × 100]

^b^Containers that were found to have a significantly greater relative proportion of use as breeding sites by *Ae. aegypti* relative to the availability of that container class (*p* < 0.001)

**Table 5:** Juvenile *Aedes aegypti* abundance classified according to material, class and capacity of collection container, type of shade cover and month and area of survey recorded between March and May 2021 in the city of Córdoba, Argentina

Collection container	Total number of juveniles collected	Number of containers surveyed	Abundance^a^
*Material*			
Plastic	281	11	6.9 ± 21.6
Glass	320	13	6.15 ± 24.32
Metal	106	5	8.83 ± 21.24
Cement	18	4	1.5 ± 2.68
Ceramic	29	1	9.67 ± 16.74
Vegetal	7	3	1.4 ± 2.07
*Class*			
Apical leaf axil	7	3	1.4 ± 2.07
Bottles	96	8	1.96 ± 10.36
Jars	76	5	7.6 ± 15.64
Buckets	4	1	2 ± 2.83
Animal water containers	7	2	1.67 ± 2.4
Dishes	86	4	4 ± 10.7
Vases	293	5	41.86 ± 57.14
Floor drains	32	6	2 ± 3.34
Plastic or tarpaulin covers	134	2	22.33 ± 49.06
Others	26	1	6.5 ± 13
*Capacity (litres)*			
Very small (< 0.5)	411	22	7.75 ± 21.14
Small (1—8)	313	9	4.47 ± 20.92
Medium (10—20)	37	6	7.74 ± 10.67
Month of survey			
March 2021	461	26	7.09 ± 19.38
April 2021	284	9	8.6 ± 30
May 2021	16	2	0.52 ± 2.25
*Shade*			
Vegetation	111	13	8.54 ± 21
Artificial	586	55	6 ± 16.2
None (sun)	318	86	3.7 ± 14.53
*Area*			
Central	359	29	12.38 ± 33.82
Southeast	144	65	2.22 ± 7.42
Southeast	258	34	7.59 ± 22.35

^a^Abundance is presented as the mean number of juvenile *Aedes aegypti* per container ± standard deviation

Juvenile *Ae. aegypti* were collected in containers of all capacities, but they were predominantly present in small and very small containers (80.4% of the total number of positive containers), which were also the most frequent container types in the peridomiciles (76.36% of all water-holding containers) (Table 4). In season 2, when the numbers of larvae and pupae were recorded, the mean, median and standard deviation observed per container were 7.75, 0 and 21.1, respectively, for very small containers; 4.47, 0 and 20.9, respectively, for small containers; and 7.4, 4 and 10.7, respectively, for medium-sized containers (Table 5).

**Fig. 3:**
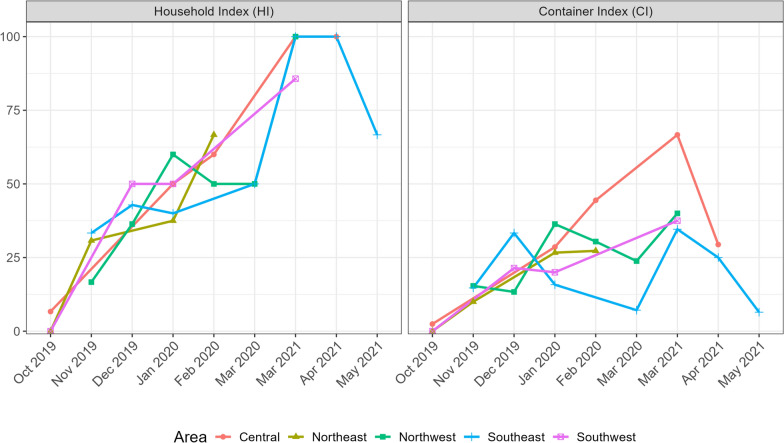
Monthly variation in the *Aedes aegypti* Household and Container indexes across the five surveyed areas of Córdoba, Argentina, between October 2019 and March 2020 and between March and May 2021

**Fig. 4:**
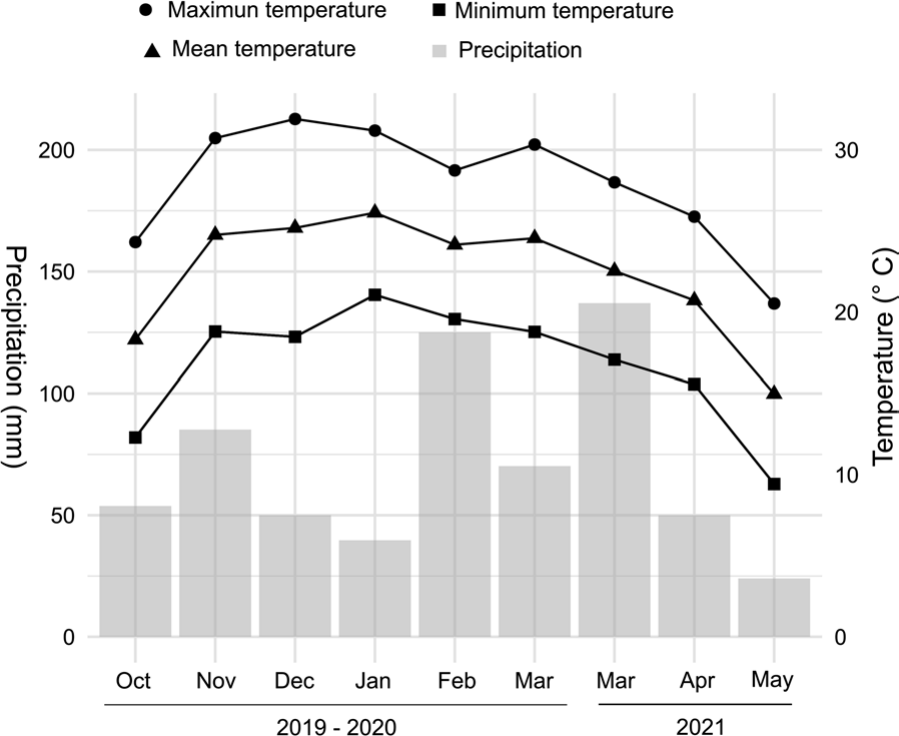
Monthly meteorological data from Córdoba city, Argentina, recorded by the National Meteorological Service between October 2019 and March 2020 and between March and May 2021

The CI and HI for* Ae. aegypti* varied between months, with the lowest CI and HI in October 2020 and the highest values in March 2021. (Fig. 3; Additional file 8: Table S5). In season 2, more juveniles were collected in March 2021 (*n* = 461) and April 2021 (*n* = 284) than in May 2021 (*n* = 16) (Table 5). Importantly, October and May mark the beginning and end of the warm and rainy summer season, respectively. The highest monthly accumulated precipitation was recorded in February 2020 and March 2021 (Fig. 4). The highest maximum daily temperature was recorded in December 2019, whereas the highest mean and minimum temperatures were recorded in January 2020. The lowest temperatures were recorded in May 2021 (Fig. 4).

Containers positive for juvenile* Ae. aegypti* were found in peridomiciles with diverse vegetation cover, ranging from completely uncovered to values of 0.59 for the tree cover proportion and 0.47 for the herb and shrub cover proportion. Approximately one-third of the containers covered by vegetation contained juvenile * Ae. aegypti* (32.53%), whereas among those covered by artificial shade or exposed to the sun, the percentages of positive containers were lower (Table 4). In terms of abundance, the containers shaded by vegetation presented with the highest mean juvenile abundance per container (8.54 ± 21) (Table 5).

**Fig. 5:**
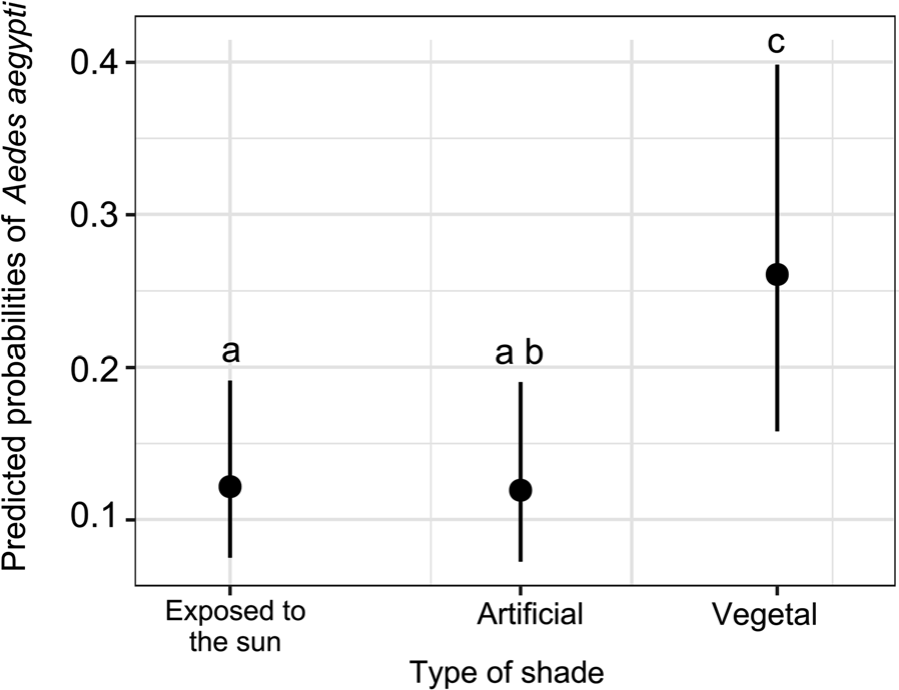
Estimated probabilities for the presence of juvenile *Aedes aegypti* in containers surveyed in the city of Córdoba, Argentina, between October 2019 and March 2020 and between March and May 2021, according to the explanatory variable “type of shade”. The black dots correspond to the estimated probability, and the black bars correspond to the standard error. Significantly different shade types, as indicated by Tukey's multiple comparison test, are marked with different lowercase letters above the standard error bar (a–c:* p* = 0.0086; b–c: *p* = 0.0301)

**Fig. 6:**
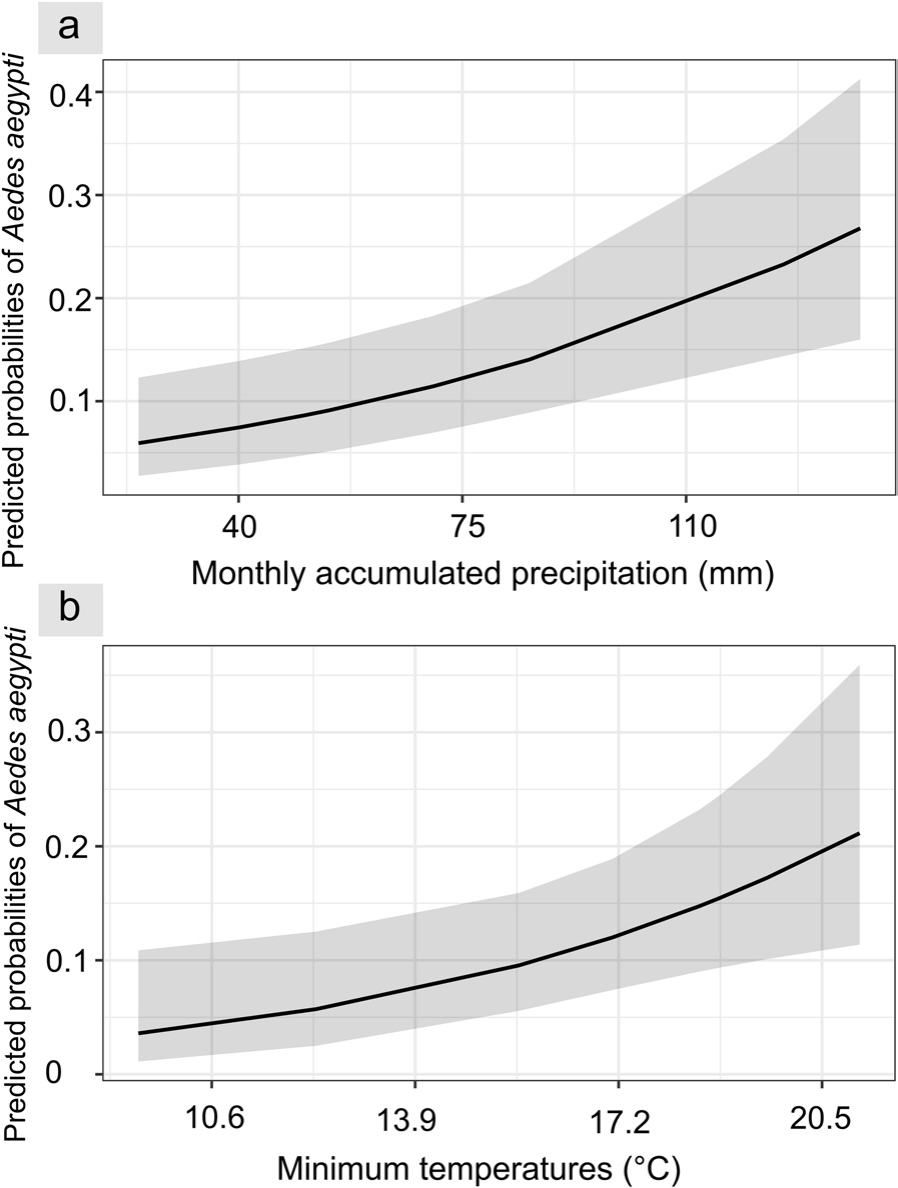
Estimated probabilities for the presence of juvenile *Aedes aegypti* in containers surveyed in the city of Córdoba, Argentina, between October 2019 and March 2020 and between March and May 2021, according to the explanatory variables.** a** Accumulated monthly precipitation,** b**: minimum temperature. The black lines correspond to the estimated probabilities for each value of the explanatory variables, and the grey shading corresponds to the 95% confidence intervals

The best-fit model explaining the presence of *Ae. aegypti* included the shade type projected at the breeding site, minimum temperature and precipitation. This model revealed that the likelihood of a container being positive for juvenile *Ae. aegypti* was 2.53-fold higher for containers shaded by vegetation [odds ratio (OR) 2.53, 95% confidence interval (CI) 1.15–5.56] than for containers exposed to the sun (Table 6). Additionally, containers shaded by vegetation were more likely  to present *Ae. aegypti* than containers under artificial shade, according to Tukey’s multiple comparison test (Fig. 5). The odds of a container containing juveniles did not differ between containers exposed to the sun and those covered by artificial shade. (Table 6; Fig. 5). In addition, the presence of juvenile *Ae. aegypti* in containers was positively associated with minimum temperatures (OR 1.75, 95% CI 1.14–2.69] and accumulated monthly precipitation (OR 1.71, 95% CI 1.27–2.31] (Table 6; Fig. 6a, b). The conditional* R*^2^ of the model was 36.1%, and the AUC value was 0.86. The random effects in the conditional model indicated that most of the variability was attributed to the "households in blocks" level (SD 0.4), whereas the variability at the household and block container level was very low (SD  9.4 x 10^–1^ and 8.78 x 10^–5^, respectively).

**Table 6:** A generalised linear mixed model used to explain the presence of juvenile *Aedes aegypti* in containers surveyed around homes in the city of Córdoba, Argentina, from October 2019 to March 2020 and March to May 2021

Variable	Odds ratio	95% Confidence interval	*p*
Intercept (container exposed to the sun)	0.14	0.08–0.23	< 0.001
Artificial shade	0.98	0.50–1.90	0.950
Shade provided by vegetation	2.53	1.15–5.56	0.021
Minimum temperature	1.75	1.14–2.69	0.013
Accumulated monthly precipitation	1.71	1.27–2.31	0.001

The best-fit model explaining the abundance of juvenile *Ae. aegypti* in containers included vegetation cover by trees, the interaction between tree cover and the type of shade, and the capacity of the container (Table 7). Although the estimated juvenile *Ae. aegypti* abundance was not different for containers exposed to different types of shade and exposed to the sun [because the 95% CI contains a rate ratio (RR) of 1], the results also showed that shade provided by artificial structures and by vegetation could increase the estimated abundance. In addition, vegetation cover by trees had a strong positive effect on the abundance of juveniles in containers that were shaded by vegetation (RR 122.52, 95% CI 6.23–2411.45). The interaction between vegetation cover by trees and artificial shade appeared to have a positive effect on the abundance of juveniles, despite the 95% CI having a RR = 1, taking into consideration the level exposed to the sun as the reference level. Increasing container capacity was positively associated with juvenile *Ae. aegypti* abundance. The conditional* R*^2^ of the model was 10.6% (Table 7).

**Table 7:** A generalised linear mixed model was used to explain the abundance of juvenile *Aedes aegypti* in containers in the city of Córdoba, Argentina, between March and May 2021

Variable	Rate ratio	95% Confidence Interval	*p*
Intercept (container exposed to the sun)	0.024	0.004–0.142	< 0.001
Artificial shade	1.48	0.2–10.81	0.70
Shade provided by vegetation	8.65	0.56–134.3	0.13
Vegetation cover by trees (VCT)	0.25	0.08–0.78	0.02
VCT × Artificial shade	2.12	0.30–14.90	0.45
VCT× Shade provided by vegetation	122.52	6.23–2411.45	0.005
Capacity of container	2.25	1.01–5	0.05

The random effects in the conditional model indicated that most of the variability was attributed to the “containers in households” level (SD 4.15), whereas the variability at the household level was very low (SD 9.16 x 10^–5^) .

## Discussion

This study investigated conditions favourable for the proliferation of juvenile *Ae. aegypti* in water-bearing containers in households in Córdoba City, Argentina, located in a temperature zone and a region of dengue fever emergence. Our findings indicate an increase in the level of infestation of *Ae. aegypti* juveniles in peridomiciles over time. During the warm and wet seasons of 2009–2010, juvenile *Ae. aegypti* were found in 5.7% of surveyed households, increasing to 15.4% in 2016–17 [29]. During our study period, from 2019–2020 and 2021, the percentage of households with juvenile *Ae. aegypti* rose further to 36.7%, which is comparable to that reported in dengue endemic countries such as Colombia [56] and Brazil [57]. Additionally, this percentage is higher than the transmission risk reference thresholds established for dengue by the Pan American Health Organisation (PAHO) [58]. Entomological surveillance is critical in Argentina to monitor *Ae. aegypti* populations amid the increasing circulation of dengue virus serotype 3 (DENV-3), following previous historical community circulation of the DENV-1 and DENV-2 serotypes. In this scenario, the PAHO [59] warns of outbreak risks.

Identifying key breeding sites of *Ae. aegypti* contributes to the design of more effective and efficient public health interventions. These key sites are locality-specific and are determined by ecological and social factors [60]. In Córdoba City, juvenile *Ae. aegypti* were found in a wide variety of water-holding containers, although plastic jars were most frequently found container type in peridomiciles. In contrast, Almirón and Asis [61] reported 20 years ago that tyres were the most common juvenile habitat. This change is possibly related to the vector control and educational campaign conducted by the Epidemiology Area of the Ministry of Health of Córdoba since the first outbreak of dengue in 2009 [62]. However, the reduced risk of tyres as a habitat for juvenile *Ae. aegypti* for did not mean the eradication of the mosquito populations; rather, it raises the possibility that mosquitoes are occupying new breeding sites. Rainfall can accumulate in the non-biodegradable plastic packaging in which most consumer goods are packaged, resulting in such packaging becoming an ideal habitat for *Ae. aegypti* once discarded [63]. Notably, vases and tarpaulins/plastic covers are highly productive larval habitats; the latter have been identified previously as atypical and important larval habitats in Peru [64]. Similar to the findings of Medeiros-Sousa et al. [65], we found juvenile *Ae. aegypti* in the leaf axils of vegetation, *Callisia fragans*, indicating their potential role as larval habitats in urban areas. In general, a few bottles and two temporary canvas pools and a partially filled pool were found with juvenile *Ae. aegypti*, which is consistent with other studies in Argentina [46, 66–68]. Our findings are consistent with the literature on *Ae. aegypti* breeding sites (e.g. [69, 70]) and indicate that this mosquito species has versatile reproductive strategies over time and can explore a wide variety of water-holding containers, whether natural or man-made.

Juvenile *Ae. aegypti* and *Cx. pipiens* were found to share water-holding containers. Mosquitoes belonging to both groups are known as opportunistic feeders, respond rapidly to environmental changes [71] and have been frequently reported to share larval habitats in other regions [26, 46, 66]. In our field work, we also reported the presence of juvenile *Cx. apicinus*, but these were never found together with juvenile *Ae. aegypti*.

We found that *Ae. aegypti* used fewer containers than were available as potential juvenile habitats. On average, 10 water-holding containers were found in each peridomicile; however, in most of the households only a single container was found to be positive for juvenile *Ae. aegypti*. In addition, the variable for the number of water-holding containers per household was not selected in the final statistical models. We expected a positive relationship between the number of positive containers and the total number of water-holding containers, as indicated by Kittayapong and Strickman [72]. Reiter [17] explained that a greater number of available water containers might favour the distribution of a small number of *Ae. aegypti* eggs among many sites. In addition, distributing offspring among several breeding sites is a strategy used by females to avoid intraspecific competition in each container [25]. The underexploitation of water-holding containers available in the houses can be explained following Abreu et al. [73], who reported that, under laboratory and seminatural conditions, the number of containers used by females to lay their eggs increased with increasing container availability (skip oviposition). However, the number of containers that were colonised tended to stabilise at approximately five, even when more water-holding containers were available. According to those authors, *Ae. aegypti* females had “preferred breeding sites”, i.e. sites of similar characteristics to others but that were selected for laying a greater number of eggs owing to the presence of conspecifics. In turn, Wong et al. [74] and Costa da Silva et al. [75] demonstrated that *Ae. aegypti* female mosquitoes can assess habitat quality by observing the reproductive success of other females. In the case of Córdoba City, Almirón et al. [76] reported that *Ae. aegypti* preferred to lay eggs in ovitraps that had intermediate egg density (40 eggs per ovitrap) rather than in ovitraps that had no eggs. Furthermore, if the selected breeding site becomes unavailable, females could disperse and colonise some of the available and still unused containers within the same peridomicile [74]. Therefore, human social factors related to water management that result in high numbers of suitable oviposition sites in neighbouring houses may result in clusters of *Ae. aegypti* in small areas [13, 14]).

*Aedes aegypti* juveniles were found to be present in containers ranging in size from very small (< 0.5 l) to extremely large (> 25,000 l). The best fit model for the abundance of juvenile *Ae. aegypti* showed a positive relationship between container capacity and abundance, in agreement with the results described by Barrera [25], Flaibani et al. [46] and David et al. [77]. In this context, there is evidence that medium and large containers (between 10 and 200 l) produce the greatest number of adults per breeding site [21] and the greatest number of adults [25, 78]. Additionally, they may be the primary source of adult mosquitoes dispersing to nearby sites [14], and they can be targeted more effectively [79]. However, small containers protect larvae from predation and interspecific competition and facilitate faster development [22]. Notably, small and very small containers were the most common juvenile *Ae. aegypti*-positive containers. The role of small containers should not be underestimated as there is evidence identifying them as highly productive sites [80].

Many previous studies have revealed the positive effects of the shade provided by vegetation on juvenile mosquito populations [25, 26, 70, 72, 78]. We found a significant positive relationship between the shade provided by vegetation and tree cover and the presence and abundance of juvenilef *Ae. aegypti*. The positive effect of vegetation shade, rather than total peridomicile cover, on *Ae. aegypti* populations could be explained by the provision of a food source in the form of detritus directly above the water, in accordance with Vezzani et al. [24] and their description of the strongest influence of vegetation cover over breeding sites. In addition, vegetation can increase mosquito reproduction, as better fed larvae give rise to larger adults [25, 78]. Another aspect that favours *Ae. aegypti* populations is that shade can regulate water temperature in containers, which is very important for larval development, as larvae are highly dependent on temperature [2]. The nonbeneficial effect of artificial shade on the containers may indicate that the containers do not need such strong protection from the sun, as Cordoba is located in a temperate zone, and water temperatures affected by irradiation may not be detrimental to juveniles.

Our findings suggest that explanatory variables may provide different information, depending on the scale of analysis. Previous neighbourhood- and city-scale studies in the city of Córdoba revealed a negative association between tree vegetation and larvae and pupae, with a greater presence of the latter in urbanised areas and near water channels [31, 33]. Benitez et al. [32] developed temporal predictive models in Córdoba with a vegetation index, such as the most important variables for egg prediction. These authors associated the increase in the vegetation index with increases in humidity and precipitation in the near past but not with effects on microscale refugia or food. In contrast, at the finer scale of peridomiciles, vegetation cover has a direct effect and is positively associated with the likelihood of the presence and higher estimated abundance of juvenile *Ae. aegypti*. Similarly, in a study conducted in the province of Buenos Aires, Vezzani et al. [24] reported that vegetation had a significant influence on microhabitat suitability at close distances (between 0.5 and 3 m) from the breeding site.

Local weather conditions (minimum temperature, precipitation) with time lags were positively related to the presence of juvenile *Ae. aegypti*, similar to the results of prior studies in Chaco, Buenos Aires and Córdoba (all in Argentina) [32, 67, 68]. Environmental variables are key factors in the modelling of *Ae. aegypti* activity. The containers most frequently harbouring *Ae. aegypti* immatures were likely filled with rainwater. Warmer minimum air temperatures were related to the presence of juvenile *Ae. aegypti*. In October, the lowest proportion of positive containers coincided with the lowest average minimum temperature (15 °C). In the following months, the proportion of positive containers and the minimum temperature increased. This result coincides with the activity threshold of 15 °C, above which higher female activity and oviposition are observed in Córdoba [32].

Entomological surveillance continues to be a fundamental strategy for the prevention of dengue in regions with increasing incidence rates [58]. The results obtained in this study highlight certain recommendations that could be useful for the prevention of *Ae. aegypti* proliferation. Among these are the identification and elimination of artificial containers, especially plastic containers, from peridomiciles. In addition, public education efforts should emphasise the risk associated with containers protected by vegetation, as well as the importance of not overlooking unconventional breeding sites. If positive containers are targeted for vector control, females that would have preferentially oviposited at those sites may instead distribute their eggs among other suitable and vacant containers. In addition, as the main breeding sites have changed over time, continued surveillance of potential juvenile habitats remains essential for effective vector control.

Studying environmental conditions at the microscale is important to ensure effective and suitable intervention management. This is the first work that has evaluated the habitat of juvenile *Ae. aegypti* under natural conditions and at the microenvironmental scale in the city of Córdoba. Containers nested in households and households nested in blocks were studied in a stratified sampling design that considered the clustering of adults and juveniles and the most accepted flight distance for adults under natural conditions, as suggested by recent studies [13, 15].

A limitation of this study was the interruption of household sampling due to the COVID-19 pandemic; thus, the following year, the surveys were conducted without the support of the Ministry of Health technicians. Moreover, during the first year of data collection, it was not possible to record the abundance of *Ae. aegypti*, as the Ministry of Health working protocol prioritised maximising the number of households visited and minimising the time spent in each one. Despite these limitations, one of the main strengths of this study was its collaboration with the Ministry of Health, which facilitated access to households throughout the city. This partnership underscores the critical importance of cross-sectoral collaboration between academic institutions and public health agencies to conduct applied research that will inform more effective vector control strategies for arboviral disease prevention.

## Conclusions

Water-bearing containers are widely available and highly diverse in terms of shape, size and material in the city of Córdoba, providing favourable conditions for the persistence of *Ae. aegypti*. Abandoned nonfunctional and cryptic sites, such as tarpaulin covers, could serve as breeding sites where eggs are able to survive the cold and dry seasons. The presence and abundance of *Ae. aegypti* are strongly influenced by microenvironmental conditions such as the shade provided by vegetation, relatively high minimum temperatures and increased precipitation. In the case of vector control measures, it should be considered that *Ae. aegypti* has a wide variety and availability of potential breeding sites in households. This, together with the favourable conditions of food and shelter provided by vegetation, might give the mosquito the opportunity to re-establish populations and persist in the same household. The results of this study are supported by generalised linear mixed models, which take into account the lack of independence between containers within the same household or block. Our findings highlight the importance of incorporating fine-scale environmental variables into vector control planning.

## Supplementary Information

**Additional file 1: Figure S1. **Land use categories (km²) of the surveillance areas defined by the *Aedes aegypti* surveillance programme of the Ministry of Health in the city of Córdoba.

**Additional file 2: Table S1.** Land use categories (km²) and demographic indicators across the surveillance areas defined by the *Aedes aegypti* surveillance programme of the Ministry of Health in the city of Córdoba.

**Additional file 3: Table S2. **Results of comparing the deviance of the models that would explain the presence of juvenile *Aedes aegypti* according to environmental and microenvironmental variables. Models with and without interactions between peridomicile vegetation cover and the type of shade projected on the container were compared with the null model (without explanatory variables) via the ANOVA function of the CAR package.

**Additional file 4: Table S3. **Results of the analysis of the model.sel function of the MuMIn package to evaluate the models that would explain the presence of juvenile *Aedes aegypti* according to environmental and microenvironmental variables.

**Additional file 7: Table S4. **Results of comparing the deviance of the models that would explain the presence of juvenile *Aedes aegypti* according to environmental and microenvironmental variables. Models with and without interactions between peridomicile vegetation cover and the type of shade projected on the container were compared with the null model (without explanatory variables) via the ANOVA function of the CAR package.

**Additional file 5: Figure S2. **Residuals diagnostic for the adequacy of the Poisson GLMM.

**Additional file 6: Figure S3. **Evaluation of zero inflation for Poisson GLMM adequacy.

**Additional file 8: Table S5. Household index per month per area registered in the City of Córdoba, Argentina, between October 2019 and March 2020 and between March and May 2021.**

**Additional file 9: Figure S4.** Frequency distribution of surveyed water containers and *Aedes aegypti* breeding sites, stratified by container class, month of survey, and area of the city of Córdoba, Argentina, for the periods October 2019–March 2020 and March–May 2021

## Data Availability

The datasets supporting the conclusions of this article are included in the article and its additional files.

## References

[1] Paixão E, Teixeira M, Rodrigues L. Zika, chikungunya and dengue: the causes and threats of new and re-emerging arboviral diseases. BMJ Glob Health. 2018;3:e000530. 10.1136/bmjgh-2017-000530. 29435366 10.1136/bmjgh-2017-000530 PMC5759716

[2] Mordecai E, Caldwell J, Grossman M, Lippi C, Johnson L, Neira M, et al. Thermal biology of mosquito-borne disease. Ecol Lett. 2019;22:1690–708. 10.1111/ele.13335. 31286630 10.1111/ele.13335 PMC6744319

[3] López M, Gómez A, Müller G, Walker E, Robert M, Estallo E. Relationship between climate variables and dengue incidence in Argentina. Environ Health Perspect. 2023;131:057008. 10.1289/ehp11616. 37224070 10.1289/EHP11616 PMC10208431

[4] Kamal M, Kenawy M, Rady M, Khaled A, Abdallah S. Mapping the global potential distributions of two arboviral vectors* Aedes aegypti* and* Ae. albopictus* under changing climate. PLoS ONE. 2018. 10.1371/journal.pone.0210122. 30596764 10.1371/journal.pone.0210122 PMC6312308

[5] Pan American Health Organisation (PAHO). Dengue: informe de situación en las Américas, semana epidemiológica 47. 2024. https://www.paho.org/sites/default/files/2024-12/2024-cde-dengue-sitrep-americas-epi-week-47-12-dec-es.pdf. Accessed 17 Dec 2024.

[6] Robert M, Tinunin D, Benitez E, Ludueña-Almeida F, Romero M, Stewart-Ibarra A, et al. Arbovirus emergence in the temperate city of Córdoba, Argentina, 2009–2018. Sci Data. 2019;6:276. 10.1038/s41597-019-0295-z. 31754110 10.1038/s41597-019-0295-z PMC6872715

[7] Ministry of Health of Córdoba. Ministeriodesalud.cba.gov.ar. 2024. Protocolo de abordaje integral de Dengue, Zika y Chikungunya 2024–2028. 2024. https://ministeriodesalud.cba.gov.ar/wp-content/uploads/2024/08/1-Protocolo-Dengue-Zika-y-Chikungunya-2024-2028.pdf. Accessed 16 Dec 2024.

[8] Soria C, Almirón W, Stewart Ibarra A, Crocco LB. Systematic review of impacts of educational interventions to control breeding sites of *Aedes aegypti* and *Aedes albopictus* mosquitoes. Am J Trop Med Hyg. 2024;110:979. 10.4269/ajtmh.23-0427. 38579697 10.4269/ajtmh.23-0427 PMC11066344

[9] Achee N, Gould F, Perkins A, Reiner R Jr, Morrison A, Ritchie S, et al. A critical assessment of vector control for dengue prevention. PLoS Negl Trop Dis. 2015;9:e0003655. 10.1371/journal.pntd.0003655. 25951103 10.1371/journal.pntd.0003655 PMC4423954

[10] Rossi G, Almirón WR. Mundo Sano. . Clave ilustrada para la identificación de larvas de mosquitos de interés sanitario encontradas en criaderos artificiales en la Argentina. 2004.https://mundosano.org/es/publicaciones/clave-ilustrada-para-la-identificacion-de-larvas-de-mosquitos-de-interes-sanitario-encontradas-en-criaderos-artificiales-en-la-argentina/. Accessed 30 Apr 2025.

[11] Dalpadado R, Amarasinghe D, Gunathilaka N. Water quality characteristics of breeding habitats in relation to the density of *Aedes aegypti* and *Aedes albopictus* in domestic settings in Gampaha district of Sri Lanka. Acta Trop. 2022;229:106339. 10.1016/j.actatropica.2022.106339. 35114170 10.1016/j.actatropica.2022.106339

[12] LaCon G, Morrison A, Astete H, Stoddard S, Paz-Soldan V, Elder J, et al. Shifting patterns of *Aedes aegypti* fine scale spatial clustering in Iquitos, Peru. PLoS Negl Trop Dis. 2014;8:e3038. 10.1371/journal.pntd.0003038. 25102062 10.1371/journal.pntd.0003038 PMC4125221

[13] Bergero PE, Ruggerio CA, Lombardo R, Schweigmann NJ, Solari HG. Dispersal of *Aedes aegypti*: field study in temperate areas using a novel method. J Vector Borne Dis. 2013;50:163–70. 24220074

[14] Schafrick N, Milbrath M, Berrocal V, Wilson M, Eisenberg J. Spatial clustering of *Aedes aegypti* related to breeding container characteristics in coastal Ecuador: implications for dengue control. Am J Trop Med Hyg. 2013;89:758–65. 10.4269/ajtmh.12-0485. 24002483 10.4269/ajtmh.12-0485 PMC3795109

[15] Moore T, Brown H. Estimating *Aedes aegypti* (Diptera: Culicidae) flight distance: meta-data analysis. J Med Entomol. 2022;59:1164–70. 10.1093/jme/tjac070. 35640992 10.1093/jme/tjac070

[16] Estallo E, Carbajo A, Grech M, Frias CM. Spatio-temporal dynamics of dengue 2009 outbreak in Córdoba City Argentina. Acta Trop. 2014;136:129–36. 10.1016/j.actatropica.2014.04.024. 24795212 10.1016/j.actatropica.2014.04.024

[17] Reiter P. Oviposition, dispersal, and survival in *Aedes aegypti*: implications for the efficacy of control strategies. Vector Borne Zoonotic Dis. 2007;7:261–73. 10.1089/vbz.2006.0630. 17627447 10.1089/vbz.2006.0630

[18] Edman J, Scott T, Costero A, Morrison A, Harrington L, Clark G. *Aedes aegypti* (Diptera: Culicidae) movement influenced by availability of oviposition sites. J Med Entomol. 1998;35:578–83. 10.1093/jmedent/35.4.578. 9701948 10.1093/jmedent/35.4.578

[19] Quintana MG, Fernández MS, Salomon OD. Distribution and abundance of Phlebotominae, vectors of leishmaniasis, in Argentina: spatial and temporal analysis at different scales. J Trop Med. 2012;1:652803. 10.1155/2012/652803. 10.1155/2012/652803 PMC3270461 22315620

[20] Rocklöv J, Dubrow R. Climate change: an enduring challenge for vector-borne disease prevention and control. Nat Immunol. 2020;21:479–83. 10.1038/s41590-020-0648-y. 32313242 10.1038/s41590-020-0648-y PMC7223823

[21] Garelli F, Espinosa M, Weinberg D, Coto H, Gaspe M, Gürtler R. Patterns of *Aedes aegypti* (diptera: culicidae) infestation and container productivity measured using pupal and Stegomyia indices in northern Argentina. J Med Entomol. 2009;46:1176–86. 10.1603/033.046.0528. 19769052 10.1603/033.046.0528 PMC3075972

[22] Qureshi A, Keen E, Brown G, Cator L. The size of larval rearing container modulates the effects of diet amount and larval density on larval development in *Aedes aegypti*. PLoS ONE. 2023. 10.1371/journal.pone.0280736. 36696416 10.1371/journal.pone.0280736 PMC9876358

[23] Vezzani D, Schweigmann N. Suitability of containers from different sources as breeding sites of *Aedes aegypti* (L.) in a cemetery of Buenos Aires City, Argentina. Mem Inst Oswaldo Cruz. 2002;97:789–92. 10.1590/S0074-02762002000600006. 12386697 10.1590/s0074-02762002000600006

[24] Vezzani D, Rubio A, Velázquez S, Schweigmann N, Wiegand T. Detailed assessment of microhabitat suitability for *Aedes aegypti* (diptera: culicidae) in Buenos Aires, Argentina. Acta Trop. 2005;95:123–31. 10.1016/j.actatropica.2005.03.010. 15993832 10.1016/j.actatropica.2005.03.010

[25] Barrera R, Amador M, Clark G. Ecological factors influencing *Aedes aegypti* (Diptera: Culicidae) productivity in artificial containers in Salinas, Puerto Rico. J Med Entomol. 2006;43:484–92. 10.1603/0022-2585(2006)43[484:efiaad]2.0.co;2. 16739405 10.1603/0022-2585(2006)43[484:efiaad]2.0.co;2

[26] Vezzani D, Albicócco A. The effect of shade on the container index and pupal productivity of the mosquitoes *Aedes aegypti* and *Culex pipiens* breeding in artificial containers. Med Vet Entomol. 2009;23:78–84. 10.1111/j.1365-2915.2008.00783.x. 19239617 10.1111/j.1365-2915.2008.00783.x

[27] Sommerfeld J, Kroeger A. Eco-bio-social research on dengue in Asia: a multicountry study on ecosystem and community-based approaches for the control of dengue vectors in urban and peri-urban Asia. Pathog Glob Health. 2012;106:428–35. 10.1179/2047773212Y.0000000055. 23318234 10.1179/2047773212Y.0000000055 PMC3541880

[28] Domínguez M, Ludueña-Almeida FF, Almirón WR. Dinámica poblacional de *Aedes aegypti* (Diptera: Culicidae) en Córdoba Capital. Rev Soc Entomol Argent. 2000;59:41–50.

[29] Estallo E, Sippy R, Stewart-Ibarra A, Grech M, Benitez E, Ludueña-Almeida FF, et al. A decade of arbovirus emergence in the temperate southern cone of South America: dengue, *Aedes aegypti* and climate dynamics in Córdoba, Argentina. Heliyon. 2020;6:e04858. 10.1016/j.heliyon.2020.e04858. 32954035 10.1016/j.heliyon.2020.e04858 PMC7489993

[30] Grech M, Sartor P, Almirón W, Ludueña-Almeida FF. Effect of temperature on life history traits during immature development of *Aedes aegypti* and *Culex quinquefasciatus* (Diptera: Culicidae) from Córdoba city, Argentina. Acta Trop. 2015;146:1–6. 10.1016/j.actatropica.2015.02.010. 25733491 10.1016/j.actatropica.2015.02.010

[31] Estallo E, Sangermano M, Grech M, Ludueña-Almeida FF, Frías-Cespedes M, Ainete M, et al. Modelling the distribution of the vector* Aedes aegypti *in a central Argentine city. Med Vet Entomol. 2018;32:451–61. 10.1111/mve.12323. 30027565 10.1111/mve.12323

[32] Benitez E, Estallo E, Grech M, Frías-Céspedes M, Almirón W, Robert M, et al. Understanding the role of temporal variation of environmental variables in predicting *Aedes aegypti* oviposition activity in a temperate region of Argentina. Acta Trop. 2021;216:105744. 10.1016/j.actatropica.2020.105744. 33189713 10.1016/j.actatropica.2020.105744

[33] Benitez E, Ludueña-Almeida FF, Frías-Céspedes M, Almirón WR, Estallo E. Could land cover influence *Aedes aegypti* mosquito populations? Med Vet Entomol. 2020;34:138–44. 10.1111/mve.12422. 31840284 10.1111/mve.12422

[34] National Meteorological Service (Servicio Metereológico Nacional). National Meteorological Service. 2025. Descarga de datos históricos. www.smn.gob.ar/descarga-de-datos. Accessed 8 Jan 2025.

[35] Provincial Census of Population (Censo Provincial de Población). datosestadistica.cba.gov.ar. 2022. Serie resultados a nivel Municipal y Comunal. Municipio de Córdoba. https://datosestadistica.cba.gov.ar/. Accessed 7 Jan 2024.

[36] Almirón WR, Ludueña-Almeida F. *Aedes aegypti* (Diptera: Culicidae) en Córdoba Argentina. Rev Soc Entomol Argent. 1998;57:27–8.

[37] Darsie R. Mosquitoes of Argentina .Part I. Keys for identification of adult females and fourth stages larvae in English and Spanish (Diptera: Culicidae). Mosq Syst. 1985;17:153–253.

[38] Branda M, Laurito M, Visintin A, Almirón W. Gonoactivity of *Culex* (*Culex*) (Diptera: Culicidae) mosquitoes during winter in temperate Argentina. J Med Entomol. 2021;58:1454–8. 10.1093/jme/tjaa295. 33479774 10.1093/jme/tjaa295

[39] Fleiss JL. Statistical methods for rates and proportions. Hoboken: John Wiley & Sons; 1973.

[40] R Core Team. R: A language and environment for statistical computing. Vienna: R Foundation for Statistical Computing; 2020. https://www.R-project.org/.

[41] RStudio Team. RStudio: Integrated Development for R. 2023. http://www.posit.co/.

[42] Wei T, Simko V, Levy M, Xie Y, Jin Y, Zemla J. Package ‘corrplot.’ Statistician. 2017;56:e24.

[43] Paradis E, Claude J, Strimmer K. APE: analyses of phylogenetics and evolution in R language. Bioinformatics. 2004;20:289–90. 10.1093/bioinformatics/btg412. 14734327 10.1093/bioinformatics/btg412

[44] Zuur A, Ieno E, Walker N, Saveliev A, Smith G. Mixed effects models and extensions in ecology with R. 1 st. New York, United States: Springer New York, NY; 2009. XXII, 574. 10.1007/978-0-387-87458-6

[45] Brooks M, Kristensen K, van Benthem K, Magnusson A, Berg C, Nielsen A, et al. glmmTMB balances speed and flexibility among packages for zero-inflated generalized linear mixed modeling. R J. 2017;9:378–400.

[46] Flaibani N, Pérez A, Barbero I, Burroni N. Different approaches to characterize artificial breeding sites of *Aedes aegypti* using generalized linear mixed models. Infect Dis Poverty. 2020;90:97–107. 10.1186/s40249-020-00705-3. 10.1186/s40249-020-00705-3 PMC7393697 32736584

[47] Forstmeier W, Schielzeth H. Cryptic multiple hypotheses testing in linear models: overestimated effect sizes and the winner’s curse. Behav Ecol Sociobiol. 2011;65:47–55. 10.1007/s00265-010-1038-5. 21297852 10.1007/s00265-010-1038-5 PMC3015194

[48] Dobson A. An introduction to generalized linear models. 2nd ed. New York:: Chapman and Hall/CRC; 2002.

[49] Fox J, Weisberg S, Price B, Adler D, Douglas B, Gabriel BB, et al. Package ‘car’. 2024. https://r-forge.r-project.org/projects/car/.

[50] Akaike H. A new look at the statistical model identification. In: Parzen E, Tanabe K, Kitagawa G, editors. Selected papers of Hirotugu Akaike Springer series in statistics. New York: Springer; 1974. p. 215–22.

[51] Bartón K. MuMIn: Multi-Model Inference.R package. 2025. https://CRAN.R-project.org/package=MuMIn

[52] Robinson D, Hayes A, Couch S. broom: convert statistical objects into tidy tibbles. 2022. https://CRAN.R-project.org/package=broom.

[53] Hartig F. Package ‘DHARMa’: residual diagnostics for hierarchical (multi-level/mixed) regression models. 2024. r-project. org/package= DHARMa. 10.32614/CRAN.package.DHARMa

[54] Barr D, Levy R, Scheepers C, Tily H. Random effects structure for confirmatory hypothesis testing: keep it maximal. J Mem Lang. 2013;68:255–78. 10.1016/j.jml.2012.11.001. 10.1016/j.jml.2012.11.001 PMC3881361 24403724

[55] Sing T, Sander O, Beerenwinkel N, Lengauer T. ROCR: visualizing classifier performance in R. Bioinformatics. 2005;21:3940–1. 10.1093/bioinformatics/bti623. 16096348 10.1093/bioinformatics/bti623

[56] Vásquez-Trujillo A, Cardona-Arango D, Segura-Cardona A, Portela-Câmara D, Alves-Honório N, Parra-Henao G. House-level risk factors for *Aedes aegypti* infestation in the urban center of Castilla la Nueva, Meta State, Colombia. J Trop Med. 2021;2021:8483236. 10.1155/2021/8483236. 34725551 10.1155/2021/8483236 PMC8557085

[57] de Moura RM, Alvarenga Monteiro Marques G, Nunes Serpa L, de Brito AM, Voltolini J, Barbosa G, et al. Density of* Aedes aegypti* and* Aedes albopictus* and its association with number of residents and meteorological variables in the home environment of dengue endemic area São Paulo Brazil. Parasit Vect. 2015;8:1–9. 10.1186/s13071-015-0703-y. 10.1186/s13071-015-0703-y PMC4336725 25890384

[58] Pan American Health Organisation (PAHO). Dengue and dengue hemorrhagic fever in the Americas: guidelines for prevention and control. 1994. www.paho.org/Spanish/HCP/HCT/VBD/arias-dengue.html. Accessed 16 Dec 2024.

[59] Pan American Health Organisation (PAHO). Alerta Epidemiológica Riesgo de brotes de dengue por la mayor circulación de DENV-3 en la Región de las Américas. 2025. https://www.paho.org/sites/default/files/2025-02/2025-feb-7-phe-epi-alerta-dengue-es-final.pdf. Accessed 5 Apr 2025.

[60] Spiegel J, Bennett S, Hattersley L, Hayden M, Kittayapong P, Nalim S, et al. Barriers and bridges to prevention and control of dengue: the need for a social–ecological approach. EcoHealth. 2005. 10.1007/s10393-005-8388-x.

[61] Almirón WR, Asís R. Índices de abundancia de larvas y pupas de *Aedes aegypti* (Diptera: Culicidae) en la Ciudad de Córdoba. Rev Fac Cien Med Univ Nac Cordoba. 2003;60:37–41. 10.31053/1853.0605.v60.n1.33715. 16724440

[62] Ortega J, Espósito S. Dengue 2009–2017: políticas públicas de salud en la provincia de Córdoba. Rev Derecho Salud. 2017;1:25–37. 10.37767/2591-3476(2017)04.

[63] Gubler D. Dengue, urbanization and globalization: the unholy trinity of the 21st century. Trop Med Health. 2011;39:3–11. 10.2149/tmh.2011-S05. 22500131 10.2149/tmh.2011-S05 PMC3317603

[64] Morrison A, Gray K, Getis A, Astete H, Sihuincha M, Focks D, et al. Temporal and geographic patterns of *Aedes aegypti* (Diptera: Culicidae) production in Iquitos, Peru. J Med Entomol. 2004;41:1123–42. 10.1603/0022-2585-41.6.1123. 15605653 10.1603/0022-2585-41.6.1123

[65] Medeiros-Sousa A, de Oliveira-Christe R, Camargo A, Scinachi C, Milani G, Urbinatti P, et al. Influence of water’s physical and chemical parameters on mosquito (Diptera: Culicidae) assemblages in larval habitats in urban parks of Sao Paulo Brazil. Acta Trop. 2020;205:105394. 10.1016/j.actatropica.2020.105394. 32070677 10.1016/j.actatropica.2020.105394

[66] Cardo M, Rosín P, Carbajo A, Vezzani D. Artificial container mosquitoes and first record of *Aedes aegypti* in the islands of the Paraná Lower Delta, Argentina. J Asia Pac Entomol. 2015;18:727–33. 10.1016/j.aspen.2015.09.002.

[67] Stein M, Oria G, Almirón WR. Principales criaderos para *Aedes aegypti* y culícidos asociados, Argentina. Rev Saude Publica. 2002;36:627–30. 10.1590/S0034-89102002000600013. 12471389 10.1590/s0034-89102002000600013

[68] Fischer S, De Majo M, Quiroga L, Paez M, Schweigmann N. Long-term spatio-temporal dynamics of the mosquito *Aedes aegypti* in temperate Argentina. Bull Entomol Res. 2017;107:225–33. 10.1017/s0007485316000869. 27876100 10.1017/S0007485316000869

[69] Kahamba N, Limwagu A, Mapua S, Msugupakulya B, Msaky D, Kaindoa E, et al. Habitat characteristics and insecticide susceptibility of *Aedes aegypti* in the Ifakara area, south-eastern Tanzania. Parasit Vectors. 2020;13:2–15. 10.1186/s13071-020-3920-y. 32033619 10.1186/s13071-020-3920-y PMC7006121

[70] Souza R, Nazare R, Argibay H, Pellizzaro M, Anjos R, Portilho M, et al. Density of *Aedes aegypti* (Diptera: Culicidae) in a low-income Brazilian urban community where dengue, Zika, and chikungunya viruses co-circulate. Parasit Vectors. 2023;16:159. 37149611 10.1186/s13071-023-05766-5 PMC10163576

[71] Santana-Martínez J, Molina J, Dussán J. Asymmetrical competition between *Aedes aegypti* and *Culex quinquefasciatus* (Diptera: Culicidae) coexisting in breeding sites. Insects. 2017;24;8:111. 10.3390/insects8040111. 29064390 10.3390/insects8040111 PMC5746794

[72] Kittayapong P, Strickman D. Distribution of container-inhabiting *Aedes larvae* (Diptera: Culicidae) at a dengue focus in Thailand. J Med Entomol. 1993;30:601–6. 10.1093/jmedent/30.3.601. 8510120 10.1093/jmedent/30.3.601

[73] Abreu F, Morais M, Ribeiro S, Eiras Á. Influence of breeding site availability on the oviposition behaviour of *Aedes aegypti*. Mem Inst Oswaldo Cruz. 2015;110:669–76. 10.1590/0074-02760140490. 26154742 10.1590/0074-02760140490 PMC4569832

[74] Wong J, Stoddard S, Astete H, Morrison A, Scott T. Oviposition site selection by the dengue vector *Aedes aegypti* and its implications for dengue control. PLoS Negl Trop Dis. 2011;5:e1015. 10.1371/journal.pntd.0001015. 21532736 10.1371/journal.pntd.0001015 PMC3075222

[75] Costa da Silva A, Cabal S, Lopez K, Boloix J, Garcia Rodriguez B, Marrero K, et al. Female *Aedes aegypti* mosquitoes use communal cues to manage population density at breeding sites. Commun Biol. 2024;7:1–18. 10.1038/s42003-024-05830-5. 38297108 10.1038/s42003-024-05830-5 PMC10830494

[76] Almirón WR, Ludueña-Almeida F, Domínguez MC. Preferencia de *Aedes aegypti* (Diptera: Culicidae) por sitios para oviposición con diferentes niveles de precolonización y exposición al sol. Rev Soc Entomol Argent. 1999;58:159–64.

[77] David M, Dantas E, Maciel-de-Freitas R, Codeço C, Prast A, Lourenço-de-Oliveira R. Influence of larval habitat environmental characteristics on Culicidae immature abundance and body size of adult *Aedes aegypti*. Front Ecol Evol. 2021;9:e626757. 10.3389/fevo.2021.626757.

[78] Tun-lin W, Burkot T, Kay B. Effects of temperature and larval diet on development rates and survival of the dengue vector *Aedes aegypti* in north Queensland, Australia. Med Vet Entomol. 2000;14:31–7. 10.1046/j.1365-2915.2000.00207.x. 10759309 10.1046/j.1365-2915.2000.00207.x

[79] Tun-Lin W, Lenhart A, Nam V, Rebollar-Téllez E, Morrison A, Barbazan P, et al. Reducing costs and operational constraints of dengue vector control by targeting productive breeding places: a multi-country non-inferiority cluster randomized trial. Trop Med Int Health. 2009;14:1143–53. 10.1111/j.1365-3156.2009.02341.x. 19624476 10.1111/j.1365-3156.2009.02341.x

[80] Hammond S, Gordon A, Lugo E, Moreno G, Kuan G, López M, et al. Characterization of *Aedes aegypti* (diptera: culcidae) production sites in urban Nicaragua. J Med Entomol. 2007;44:851–60. 10.1603/0022-2585(2007)44[851:coaadc]2.0.co;2. 17915519 10.1603/0022-2585(2007)44[851:coaadc]2.0.co;2

