# Supplementary material for: Habitat characteristics that favour the presence of Aedes aegypti (Diptera: Culicidae) in households in the city of Córdoba, a temperate area of Argentina
Source: Parasit Vectors. 2025 Nov 25;18:487. doi: 10.1186/s13071-025-07114-1 (PMC12645701; doi:10.1186/s13071-025-07114-1)
Supplement: Supplementary file 2 — Additional file 2: Table S1. Land use categories (km²) and demographic indicators across the surveillance areas defined by the Aedes aegypti surveillance programme of the Ministry of Health in the city of Córdoba. [file 13071_2025_7114_MOESM2_ESM.docx]

| Response variable | Models | Explanatory variables | AIC | Loglik | deviance | Chi-square test | *df* | *P* |
| --- | --- | --- | --- | --- | --- | --- | --- | --- |
| Presence of *Aedes aegypti* larvae and/or pupae in containers | mnull | None | 489 | -241 | 481 |  |  |  |
|  | m1 | Vegetation cover by herbs and shrubs + Vegetation cover by trees + Capacity + Number of water containers + Shade + Minimum temperature + Precipitation | 470 | -224 | 448 | 33.6 | 7 | 2e-05 |
|  | m11 | Vegetation cover by herbs and shrubs* Shade + Vegetation cover by trees* Shade + Capacity + Number of water containers + Minimum temperature + Precipitation | 474 | -222 | 444 | 3.6 | 4 | 0.46 |
