# Supplementary material for: Habitat characteristics that favour the presence of Aedes aegypti (Diptera: Culicidae) in households in the city of Córdoba, a temperate area of Argentina
Source: Parasit Vectors. 2025 Nov 25;18:487. doi: 10.1186/s13071-025-07114-1 (PMC12645701; doi:10.1186/s13071-025-07114-1)
Supplement: Supplementary file 3 — Additional file 3: Table S2. Results of comparing the deviance of the models that would explain the presence of juvenile Aedes aegypti according to environmental and microenvironmental variables. Models with and without interactions between peridomicile vegetation cover and the type of shade projected on the container were compared with the null model (without explanatory variables) via the ANOVA function of the CAR package. [file 13071_2025_7114_MOESM3_ESM.docx]

| Response variable | Models | gl | AIC | ΔAIC | Weight | Explanatory variables |
| --- | --- | --- | --- | --- | --- | --- |
| Presence of *Aedes aegypti* larvae and/or pupae in containers | m1 | 11 | 470 | 0.00 | 0.69 | Vegetation cover by herbs and shrubs + Vegetation cover by trees + Capacity + Shade + Number of water containers + Minimum temperature + Precipitation |
|  | m2 | 11 | 472 | 2.16 | 0.23 | Vegetation cover by herbs and shrubs + Vegetation cover by trees + Capacity + Shade + Number of water containers +Maximum temperature + Precipitation |
|  | m3 | 11 | 474 | 4.32 | 0.08 | Vegetation cover by herbs and shrubs + Vegetation cover by trees + Capacity + Shade + Number of water containers + Mean temperature + Precipitation |
|  | mnull | 4 | 489 | 19.18 | 0.00 | None |
