# Supplementary material for: Habitat characteristics that favour the presence of Aedes aegypti (Diptera: Culicidae) in households in the city of Córdoba, a temperate area of Argentina
Source: Parasit Vectors. 2025 Nov 25;18:487. doi: 10.1186/s13071-025-07114-1 (PMC12645701; doi:10.1186/s13071-025-07114-1)
Supplement: Supplementary file 4 — Additional file 4: Table S3. Results of the analysis of the model.sel function of the MuMIn package to evaluate the models that would explain the presence of juvenile Aedes aegypti according to environmental and microenvironmental variables. [file 13071_2025_7114_MOESM4_ESM.docx]

| Response variable | Models | Explanatory variables | AIC | Loglik | deviance | Chi-square test | *df* | *P* |
| --- | --- | --- | --- | --- | --- | --- | --- | --- |
| Abundance of *Aedes aegypti* larvae and/or pupae in containers | pmnull | None | 3 | 419 | 413 |  |  |  |
|  | pm1 | Vegetation cover by herbs and shrubs + Vegetation cover by trees + Capacity + Number of water containers + Shade + Minimum temperature + Precipitation | 10 | 423 | 403 | 9.22 | 7 | 0.24 |
|  | pm11 | Vegetation cover by herbs and shrubs* Shade + Vegetation cover by trees* Shade + Capacity + Number of water containers + Minimum temperature + Precipitation | 14 | 422 | 394 | 9.84 | 4 | 0.04 |
