# Supplementary material for: Habitat characteristics that favour the presence of Aedes aegypti (Diptera: Culicidae) in households in the city of Córdoba, a temperate area of Argentina
Source: Parasit Vectors. 2025 Nov 25;18:487. doi: 10.1186/s13071-025-07114-1 (PMC12645701; doi:10.1186/s13071-025-07114-1)
Supplement: Supplementary file 5 — Additional file 7: Table S4. Results of comparing the deviance of the models that would explain the presence of juvenile Aedes aegypti according to environmental and microenvironmental variables. Models with and without interactions between peridomicile vegetation cover and the type of shade projected on the container were compared with the null model (without explanatory variables) via the ANOVA function of the CAR package. [file 13071_2025_7114_MOESM5_ESM.docx]

| Area | Month | N° of households surveyed | Household index |
| --- | --- | --- | --- |
| Central | October 2019 | 15 | 6.67 |
| NE | October 2019 | 14 | 0.00 |
| SO | October 2019 | 6 | 0.00 |
| NE | November 2019 | 15 | 33.33 |
| NO | November 2019 | 10 | 10.00 |
| SE | November 2019 | 13 | 33.33 |
| NO | December 2019 | 16 | 31.25 |
| SE | December 2019 | 8 | 50.00 |
| SO | December 2019 | 9 | 55.56 |
| Central | January 2020 | 5 | 20.00 |
| NE | January 2020 | 8 | 37.50 |
| NO | January 2020 | 6 | 50.00 |
| SE | January 2020 | 6 | 33.33 |
| SO | January 2020 | 5 | 40.00 |
| Central | February 2020 | 14 | 42.86 |
| NE | February 2020 | 8 | 25.00 |
| NO | February 2020 | 7 | 57.14 |
| NO | March 2020 | 8 | 50.00 |
| SE | March 2020 | 6 | 50.00 |
| Central | March 2021 | 2 | 100.00 |
| NO | March 2021 | 2 | 50.00 |
| SE | March 2021 | 4 | 100.00 |
| SO | March 2021 | 7 | 75.00 |
| Central | April 2021 | 4 | 80.00 |
| NE | April 2021 | 2 | 100.00 |
| SE | April 2021 | 2 | 50.00 |
| SE | May 2021 | 8 | 66.67 |

Missing areas by month correspond to households where residents did not provide consent to inspect their backyards.
