# Supplementary figures and images for: Habitat characteristics that favour the presence of Aedes aegypti (Diptera: Culicidae) in households in the city of Córdoba, a temperate area of Argentina
Source: Parasit Vectors. 2025 Nov 25;18:487. doi: 10.1186/s13071-025-07114-1 (PMC12645701; doi:10.1186/s13071-025-07114-1)

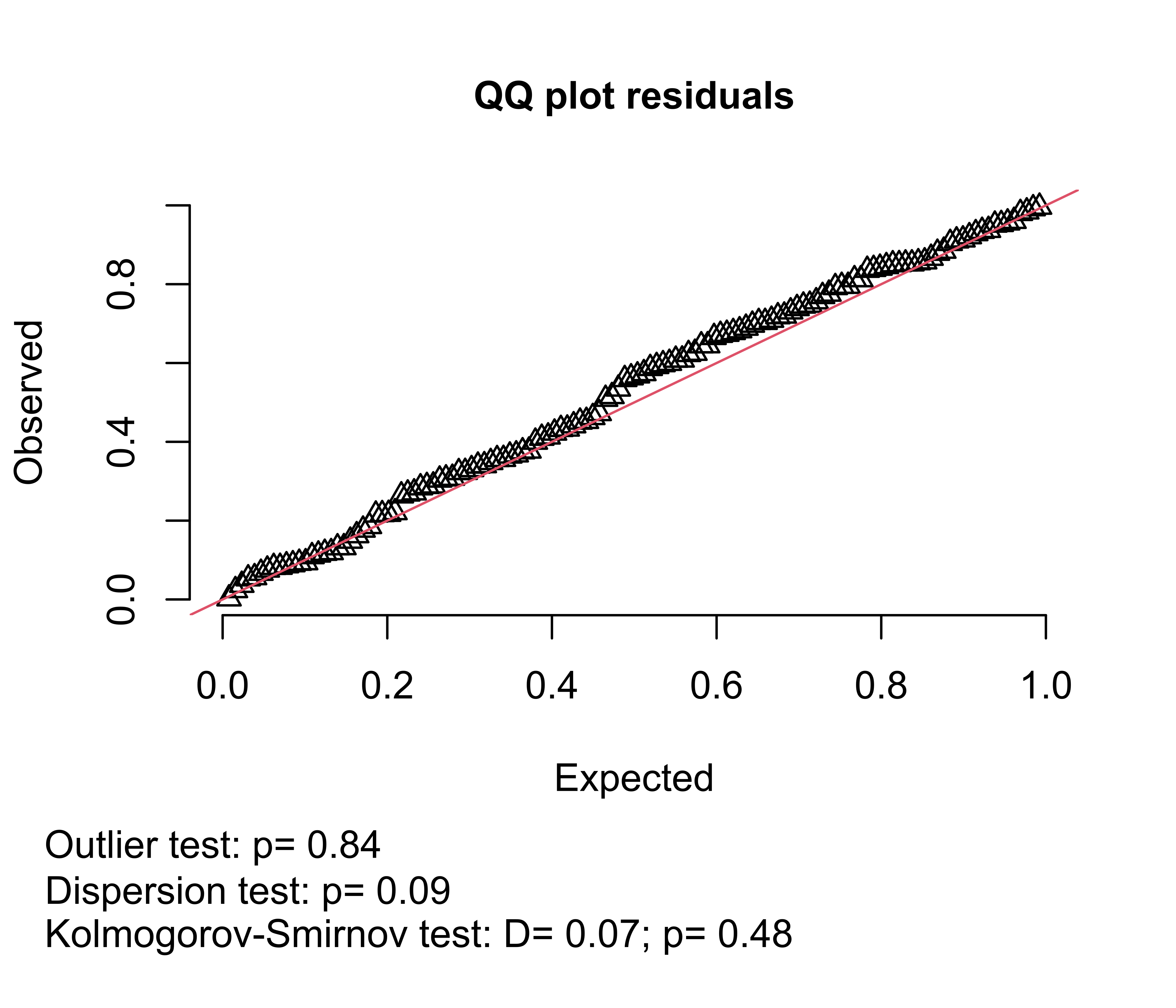

Supplement: Supplementary file 6 — Additional file 5: Figure S2. Residuals diagnostic for the adequacy of the Poisson GLMM. [file 13071_2025_7114_MOESM6_ESM.tiff]

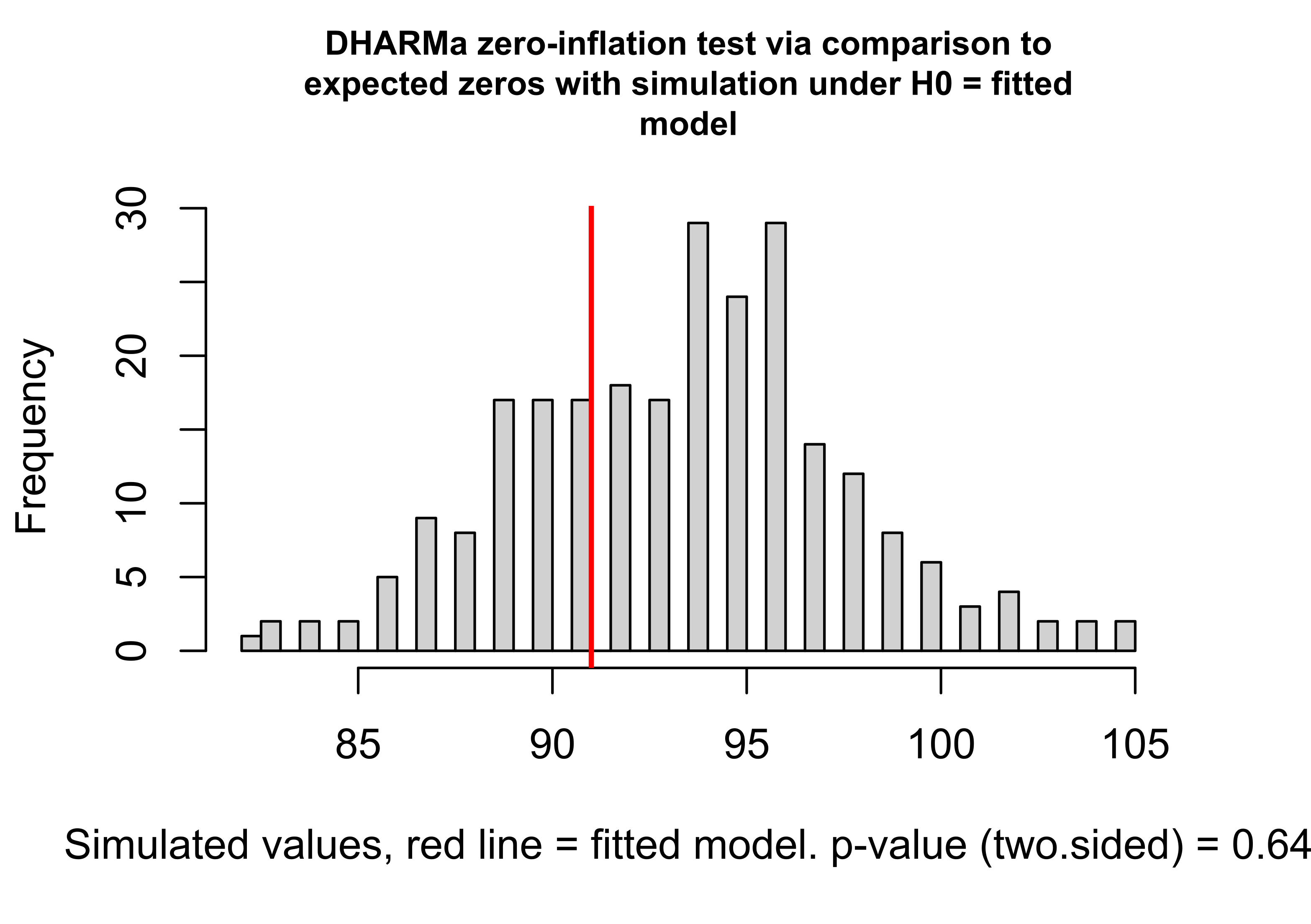

Supplement: Supplementary file 7 — Additional file 6: Figure S3. Evaluation of zero inflation for Poisson GLMM adequacy. [file 13071_2025_7114_MOESM7_ESM.tiff]

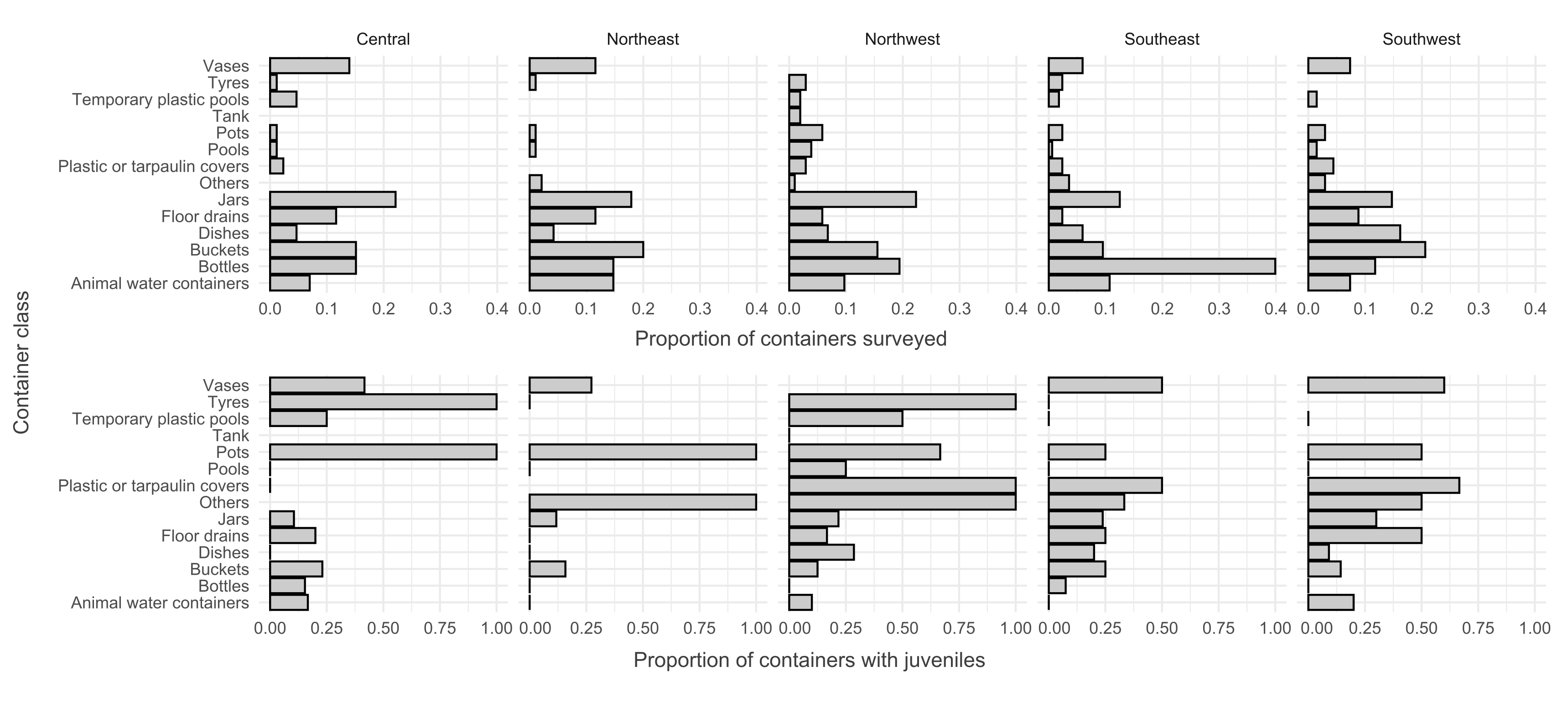

Supplement: Supplementary file 9 — Additional file 9: Figure S4. Frequency distribution of surveyed water containers and Aedes aegypti breeding sites, stratified by container class, month of survey, and area of the city of Córdoba, Argentina, for the periods October 2019–March 2020 and March–May 2021 [file 13071_2025_7114_MOESM9_ESM.tiff]
